# Burden of Nutritional Deficiencies in China: Findings from the Global Burden of Disease Study 2019

**DOI:** 10.3390/nu14193919

**Published:** 2022-09-21

**Authors:** Liyuan Han, Tian Zhao, Ruijie Zhang, Yanhua Hao, Mingli Jiao, Qunhong Wu, Jingjing Liu, Maigeng Zhou

**Affiliations:** 1 Key Laboratory of Diagnosis and Treatment of Digestive System Tumors of Zhejiang Province, Hwa Mei Hospital, University of Chinese Academy of Sciences, Ningbo 315000, China; 2 Department of Global Health, Ningbo Institute of Life and Health Industry, University of Chinese Academy of Sciences, Ningbo 315000, China; 3 Department of Health Policy, Health Management College, Harbin Medical University, Harbin 150081, China; 4 Department of Social Medicine, School of Public Health, Harbin Medical University, Harbin 150000, China; 5 National Center for Chronic and Noncommunicable Disease Control and Prevention, Chinese Center for Disease Control and Prevention, Beijing 100050, China

**Keywords:** nutritional deficiencies, GBD, trend, incidence, disability-adjusted life-years

## Abstract

From 1990 to 2019, the age-standardized incidence rate of nutritional deficiencies in China remained stable. However, the age-standardized disability-adjusted life-years (DALY) rate of nutritional deficiencies decreased from 1990 to 2019. Data were extracted from the GBD 2019 datasets. Estimated annual percentage changes (EAPCs) were calculated to assess the incidence rate, and DALY trends of nutritional deficiencies. Measures were stratified by subtypes, regions, and age groups. In 2019, the age-standardized DALY rates of dietary iron deficiency and protein-energy malnutrition reached their highest levels. The main population groups with protein-energy malnutrition and dietary iron deficiency were adults over the age of 70 and children under the age of five. The latter group also had a greater burden of vitamin A deficiency. Zhejiang, Beijing, and Guangdong reported the highest age-standardized incidence rates of nutritional deficiencies, which mainly pertained to protein-energy malnutrition and vitamin A deficiency. Tibet, Xinjiang, and Hainan had the highest age-standardized DALY rates of nutritional deficiencies, which mainly pertained to dietary iron deficiency and protein-energy malnutrition.

## 1. Introduction

China’s national levels of nutrition and general health have improved significantly in recent years. Nonetheless, important issues of undernutrition and overnutrition have not been fully resolved. For instance, China’s population faces a heavy burden of nutritional deficiencies, especially deficiencies in iron and other essential micronutrients [1].

The Sustainable Development Goals (SDGs) released by the United Nations General Assembly in 2015 aim to eliminate all forms of malnutrition by 2030. These SDGs also aim to achieve international targets relating to the stunting and wasting of children under the age of five by 2025, and to address the nutritional needs of adolescent girls, pregnant women, breastfeeding women, and older adults [2]. The Chinese government has released a new National Nutrition Plan (2017–2030) with three key aims: (i) to reduce the rate of anemia in its national population, especially among children below the age of five, pregnant women, and older adults; (ii) to reduce the rate of growth retardation in children; and (iii) to improve the rate of inpatient nutrition screening and nutrition treatment for individuals found to be malnourished [3].

Comprehensive data on the burden of nutritional deficiencies is currently lacking at the national and provincial levels. In close collaboration with the Institute for Health Metrics and Evaluation at the University of Washington in Seattle, the Chinese Center for Disease Control and Prevention (CDC) participated in the Global Burden of Diseases, Injuries, and Risk Factors Study 2019 (GBD 2019). We conducted a new, comprehensive assessment of morbidity, mortality, and disability patterns from 1990 to 2019 at both the national and provincial levels. Here we aimed to assess the burden of nutritional deficiencies, specifically by reporting trends in the incidence estimates and disability-adjusted life years (DALYs) associated with nutritional deficiencies in China over a 20-year period.

## 2. Methods

### 2.1. Framework of the GBD 2019 Study

GBD 2019 has provided consistent and updated global, regional, and national estimates of the burden of diseases, injuries, and risk factors (by integrating all available data). It makes use of all recently available data from epidemiological studies and optimizes standardized methods for the comparative assessment of 369 diseases and 87 risk factors in 204 countries and territories. Specific details of the methods used in the GBD 2019 study have been published elsewhere [4].

The study was approved by the National Center for Chronic and Noncommunicable Disease Control and Prevention. The requirement for informed consent was waived because no identifiable information was included in the analyses. The GBD 2019 study followed the Guidelines for Accurate and Transparent Health Estimates Reporting.

### 2.2. Data Sources of the China Study

The original data on nutritional deficiency in China estimated by GBD 2019 were derived from the Cause-of-Death Reporting System of the Chinese CDC, the Disease Surveillance Points system, a national survey, life registration data, hospital data, data on cancer registration in the general population, demographic and census data, as well as published data on disease incidences, illness, death, disease courses, and remission. Data on the causes of death by province were derived primarily from surveillance systems and surveys, the China Cancer Registry, and the Cause of Death Reporting System of the Chinese CDC. In this study, a total of 33 province-level administrative units were analyzed; this included 31 mainland provinces, municipalities, and autonomous regions, as well as the special administrative regions of Hong Kong and Macao.

Nutritional deficiencies were identified based on the *International Classification of Diseases and Injuries*, 10th revision (ICD-10), using the codes D50–D53·9, E00–E02, E40–E46·9, E50–E61·9, E63–E64·9, and Z13·2–Z13·3. The subcategories included protein-energy malnutrition (codes E40–E46·9 and E64·0), iodine deficiencies (codes E00–E02), vitamin A deficiencies (codes E50–E50·9 and E64·1), dietary iron deficiencies (codes D50–D50·9), and other nutritional deficiencies (codes D51–D53·9, E51–E61·9, E63–E64, and E64·2–E64·9).

### 2.3. Measures

We measured two parameters associated with nutritional deficiencies: incidence and DALYs. “Incidence” referred to the number of new cases of a given cause during a given period in a specified population. “DALYs” were derived by summing up the years of life lost due to premature mortality and the years of life lived with disability; thus, DALYs incorporated both fatal and nonfatal burdens.

We estimated the annual percentage changes in the age-standardized incidence and DALY rates to identify the trends in these two parameters of nutritional deficiency. The age-standardized incidence rate represents the number of new cases per 100,000 people, while the age-standardized DALY rate represents the years of life lived with disability and the years of life lost due to premature death per 100,000 people.

### 2.4. Statistical Analysis

The incidence of nutritional deficiency, its main subcategories, and the resulting DALYs were examined at the national and provincial levels in China.

We calculated the age-standardized rates to minimize the differences among populations with different compositions. We used a global standard—the World Health Organization (WHO) 2000–2025—to calculate the age-standardized rates according to the following equation [5]:

(1) $$\mathrm{Age}-\mathrm{standardized}\;\mathrm{rate}=\frac{\sum_{i=1}^{A}a_{1\mathrm{wi}}}{\sum_{i=1}^{A}\mathrm{wi}},$$

where and represent the age-specific rates and the number of people (or weight), respectively, in the same age subgroup of the chosen reference standard population (where i denotes the ith age class).

Log-linear regression models were used to describe the all-age and age-specific trends in the age-standardized rates. The trend results are presented as the estimated annual percentage changes (EAPCs) [6] and calculated using the following formulas:

y=a+bx +∈andEAPC = 100 × (exp (β) − 1),

where y = ln (age-standardized rate), x is the calendar year, and β is the estimated value of the slope. The above EAPC formula was then applied to calculate the 95% confidence interval, and the standard error was obtained from the fitted regression line. If the estimation of the EAPC and the lower boundary of its 95% CI were both > 0, the age-standardized rate was considered to be increasing. In contrast, if the estimation of the EAPC and the upper boundary of its 95% CI were both < 0, the age-standardized rate was considered to be decreasing. Otherwise, the age-standardized rate was considered to be stable over time.

All data were analyzed using SAS 9·4 statistical software (SAS Institute Inc., Cary, NC, USA) and R version 3.3.

## 3. Results

### 3.1. China’s Burden of Overall Nutritional Deficiency from 1990 to 2019

**Figure 1:**
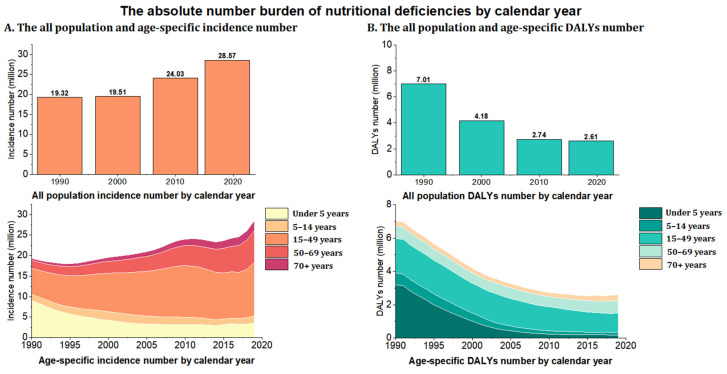
The absolute number of nutritional deficiencies from 1990 to 2019 in China. (**A**) Incidence; (**B**) DALYs. DALYs = disability-adjusted life-years.

Table 1 and Figure 1 present the incidence and DALYs numbers, their age-standardized rates, and the EAPCs in the age-standardized rates of nutritional deficiencies in China’s population from 1990 to 2019. In 2019, 28.57 million people had nutritional deficiencies, while 2.61 million people had DALYs from nutritional deficiencies in China. People aged between 15 and 49 constituted the largest proportion of cases of nutritional deficiencies and DALYs from nutritional deficiencies, at 54.92% (12.88 million cases) and 44.44% (1.45 million cases), respectively.

**Figure 2:**
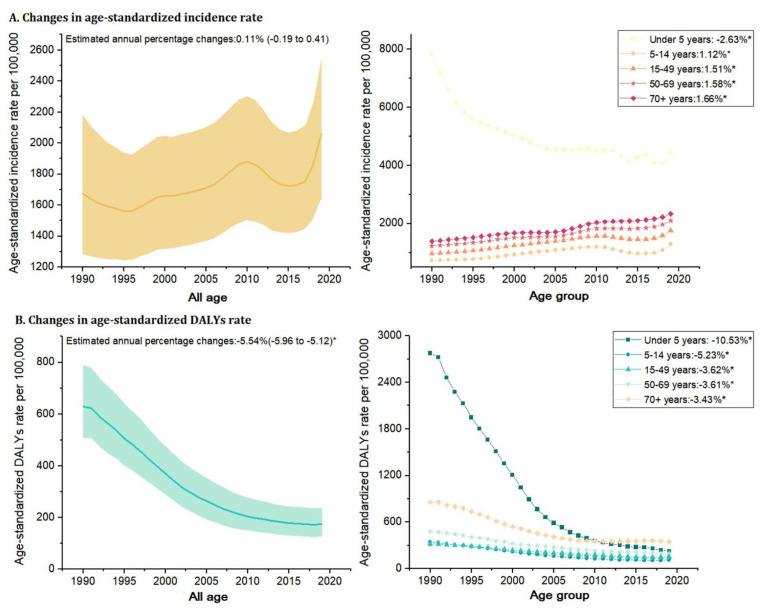
Change in the age-standardized incidence rate and DALYs rate of nutritional deficiencies from 1990 to 2019 in China. (**A**) Age-standardized incidence rate; (**B**) Age-standardized DALYs rate. DALYs = disability-adjusted life-years. Note: (*) Indicates statistically significant trend (*p* < 0.05).

The age-standardized incidence rate of nutritional deficiencies remained stable from 1990 to 2019 [EAPC: 0.11 (−0.19 to 0.41)]. The age-standardized DALY rate decreased significantly over the same period, by an average EAPC of −5.54 (−5.96 to −5.12); this corresponded to a decrease from 629.02 cases per 100,000 people to 174.78 cases per 100,000 people (Table 1 and Figure 2A).

In 2019, children under the age of five had the highest estimated age-standardized incidence rate (4414.22 per 100,000); however, the EAPC in this age group decreased between 1990 and 2019 (EAPC: −2.63 (−3.10 to −2.16)). From 1990 to 2019, the age-standardized incidence rate of overall nutrition deficiency increased significantly in people aged 15–49 (EAPC: 1.51 (1.13 to 1.90)), 50–69 (EAPC: 1.58 (1.35 to 1.80)), and over 70 (EAPC: 1.66 (1.46 to 1.87)) (Table 1 and Figure 2A). In 2019, people aged over 70 (345.89 per 100,000) had the highest rate of DALYs from overall nutritional deficiency. Nonetheless, the rate of DALYs from overall nutritional deficiency declined between 1990 and 2019 in specific age groups, especially in children under the age of five (EAPC: −10·53 [−11.00 to −10.06]) (Table 1 and Figure 2B).

### 3.2. China’s Burden of Nutritional Deficiency from 1990 to 2019 by Subcategories of Nutritional Deficiency

**Figure 3:**
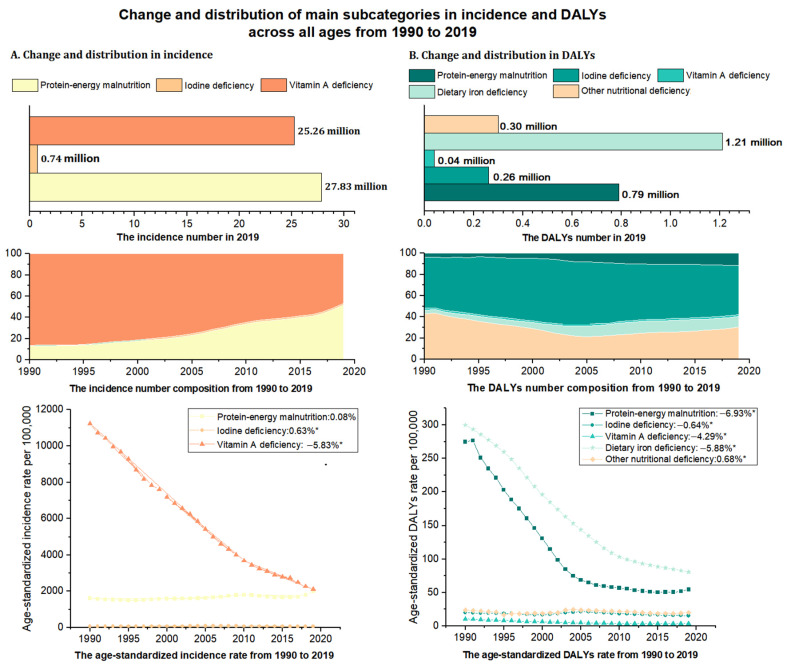
Change and distribution of main subcategories in incidence and DALYs rate in all population from 1990 to 2019 in China. (**A**) Incidence; (**B**) DALYs. DALYs = disability-adjusted life-years. Note: (*) Indicates statistically significant trend (*p* < 0.05).

Notwithstanding the limited data for some subcategories of nutritional deficiency (e.g., iron), among the main subcategories of nutritional deficiency, vitamin A deficiency had the highest age-standardized incidence rate (2113.34 per 100,000) in 2019, followed by protein-energy malnutrition (1996.51 per 100,000) and iodine deficiency (62.40 per 100,000) (Table 1 and Figure 3A). From 1990 to 2019, the age-standardized incidence rate of vitamin A deficiency declined significantly (EAPC: −5.83 (−6.17 to −5.48)), but that of iodine deficiency showed a slight increase (EAPC: 0.63 (0.10 to 1.16)) (Table 1 and Figure 3A). In 2019, dietary iron deficiency had the highest age-standardized rate of DALYs (80.69 per 100,000), followed by protein-energy malnutrition (54.57 per 100,000) and other nutritional deficiencies (20.26 per 100,000). Between 1990 and 2019, the age-standardized rates of DALYs decreased for all deficiency subcategories except other nutritional deficiency, with the largest decrease observed in protein-energy malnutrition (EAPC: −6.93 (−7.65 to −6.20)) (Table 1 and Figure 3B).

In 2019, regardless of age, protein-energy malnutrition and vitamin A deficiency had the highest age-standardized incidence rates, while protein-energy malnutrition and dietary iron deficiency had the highest age-standardized DALY rates. Comparing across age groups, the highest age-standardized incidence rate of protein-energy malnutrition was observed in children below the age of five, while the highest age-standardized incidence rate of vitamin A deficiency was observed in children aged 5–14. The highest age-standardized rate of DALYs from protein-energy malnutrition was observed in adults over the age of 70, while the highest age-standardized rate of DALYs from dietary iron deficiency was observed in children below the age of five and adults over the age of 70.

From 1990 to 2019, the age-standardized incidence rate of protein-energy malnutrition decreased only in children below the age of five; it increased in all other age groups, and showed the largest increase in adults over the age of 70. The age-standardized incidence rate of vitamin A deficiency decreased across all age groups and showed the smallest decrease in children aged 5–14. The age-standardized rates of DALYs from protein-energy malnutrition and dietary iron deficiency decreased in all age groups. The largest and smallest decreases in protein-energy malnutrition were observed in children below the age of five and adults aged 15–49, respectively. The smallest decrease in dietary iron deficiency was observed in children below the age of five. Additionally, among children below the age of five, vitamin A deficiency and protein-energy malnutrition showed the greatest reductions (out of all nutritional deficiencies) in terms of the age-standardized incidence and DALY rates, respectively.

### 3.3. Burden of Overall Nutritional Deficiency from 1990 to 2019 at the Provincial Level

**Figure 4:**
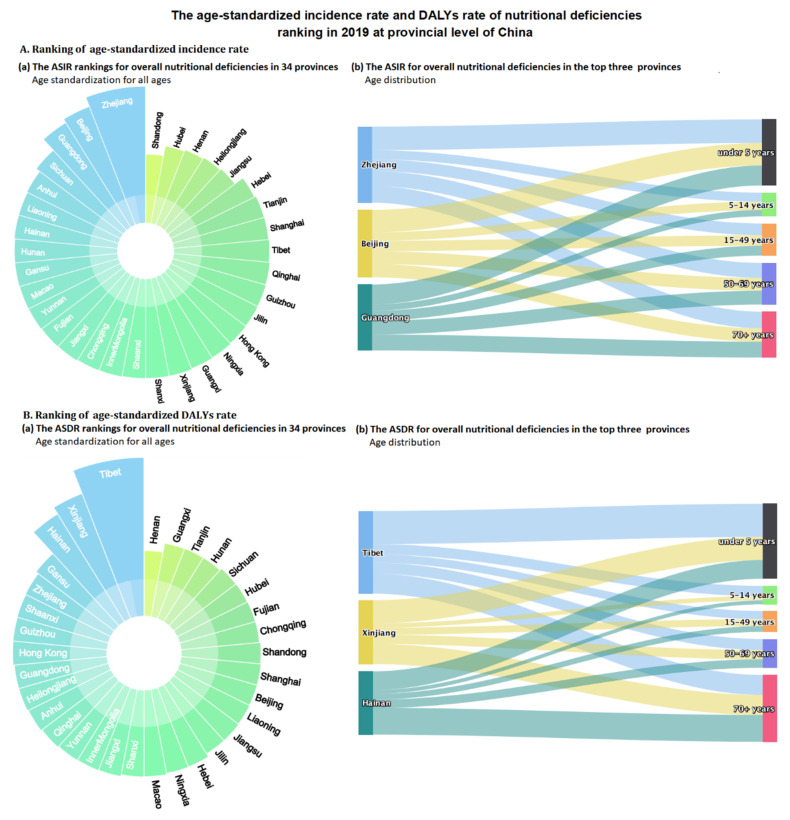
The age-standardized incidence rate and DALYs rate of nutritional deficiencies ranking in 2019 at provincial levels of China. (**A**) Age-standardized incidence rate; (**B**) Age-standardized DALYs rate. DALYs = disability-adjusted life-years.

At the provincial level, the highest age-standardized incidence rates of overall nutritional deficiency in 2019 were in Zhejiang (3443.36 per 100,000), Beijing (3048.59 per 100,000), and Guangdong (2892.1 per 100,000), whereas the lowest rate was observed in Shandong (1190.5 per 100,000) (Figure 4A and Table S1 p2). From 1990 to 2019, 14 provinces showed increases in the age-standardized incidence rate of overall nutritional deficiency, especially Liaoning (EAPC: 1.24 (1.11 to 1.37)), Zhejiang (1.21 (1.08 to 1.35)), and Hunan (1.13 (1.03 to 1.23)). The only provinces to show decreases in the age-standardized incidence rate of overall nutritional deficiency during this period were Hainan and Shanghai, which reported average EAPCs of −0.58 (−0.88 to −0.28) and −0.38 (−0.64, −0.11), respectively (Table S1 p2).

In 2019, the highest age-standardized rates of DALYs from overall nutritional deficiency were reported in Tibet (447.58 per 100,000), Xinjiang (342.32 per 100,000), and Hainan (312.91 per 100,000), and the lowest rates were reported in Henan (123.39 per 100,000) (Figure 4B and Table S2 p10). The age-standardized rates of DALYs from overall nutritional deficiency decreased in all provinces from 1990 to 2019. The most pronounced decrease was observed in Guizhou (EAPC: −7.59 (−8.07 to −7.10)). The smallest decreases were observed in the Special Administrative Regions of Hong Kong (EAPC: −1.68 (−1.90 to −1.47)) and Macao (EAPC: −2.30 (−2.43 to −2.16)), as well as in Beijing (EAPC: −2.51 (−2.85 to −2.16)) (Table S2 p10).

**Figure 5:**
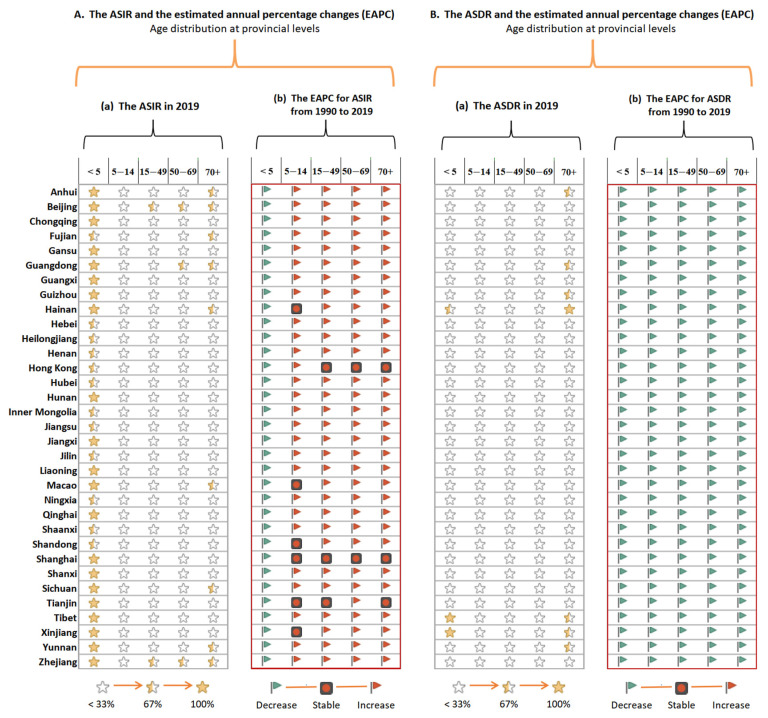
The age-standardized incidence rate and DALYs rate of overall nutritional deficiencies in 2019 and their estimated annual percentage changes (EAPC) from 1990 to 2019 by ages at provincial levels of China.

Across the age groups, the highest incidence of overall nutritional deficiency was observed in children below the age of five, especially in Zhejiang (6027.12 per 100,000). This occurred despite the decline in the age-standardized incidence rate of overall nutritional deficiency in all provinces between 1990 and 2019. The population in Guizhou reported the most rapid increases in the age-standardized incidence rates of overall nutritional deficiency in the 15–49, 50–69, and 70+ age groups, which showed annual average increments of 2.74%, 2.80%, and 2.80% respectively (Figure 5A and Table S1 p2).

Except for Tibet (1187.45 per 100,000) and Xinjiang (835.91 per 100,000), where the highest rates of DALYs from overall nutritional deficiency were observed in children below the age of five, the highest rates in other provinces were observed in adults above the age of 70, especially those in Hainan (964.54 per 100,000). The age-standardized rate of DALYs from overall nutritional deficiency declined in all provinces between 1990 and 2019 (Figure 5B and Table S2 p10).

### 3.4. Burden of Subcategories of Nutritional Deficiency from 1990 to 2019 at the Provincial Level

**Figure 6:**
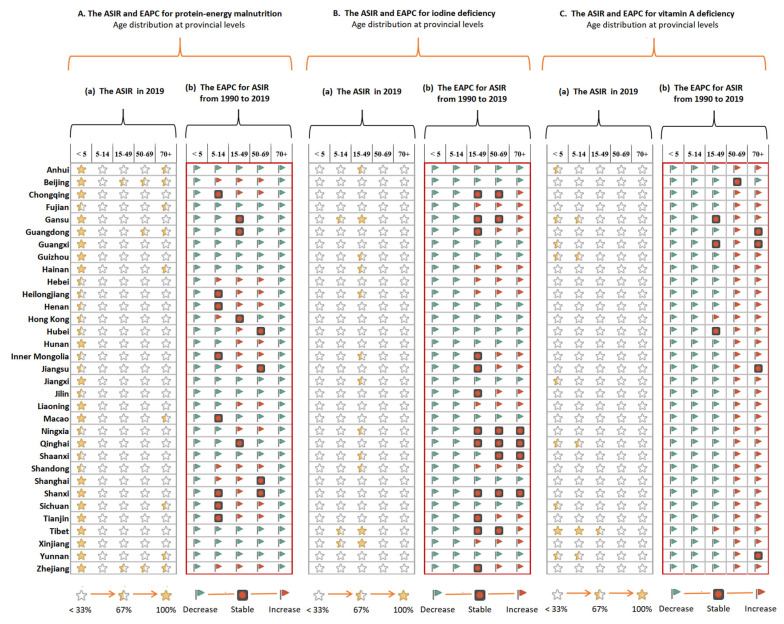
The age-standardized incidence rate for main subcategories in 2019 and their estimated annual percentage changes (EAPC) from 1990 to 2019 by ages at provincial levels of China.

Figure 6 and Tables S1 and S2 present data on the age-specific burdens and temporal trends in the subcategories of nutritional deficiencies from 1990 to 2019. In 2019, children under the age of five had the highest incidence of vitamin A deficiency in 20 provinces, especially in Tibet (13,200.38 per 100,000) (Table S1 p2). Furthermore, the highest overall rates of DALYs from dietary iron deficiency were reported in 26 provinces, especially in Tibet and in children below the age of five (453.38 per 100,000) (Table S2 p10).

There were also marked differences in the spatial distributions of the age-specific incidences and rates of DALYs across different nutritional deficiency subcategories. From 1990 to 2019, the incidences of all nutritional deficiency subcategories in children below the age of five declined. However, the increasing incidences of protein-energy malnutrition, iodine deficiency, and vitamin A deficiency were widely distributed among adults in the 15–49 (17 provinces), 50+ (33 provinces), and 70+ (16 provinces) age groups (Table S1 p2). The rates of DALYs from vitamin A deficiency and other nutritional deficiencies increased in almost all provinces (Table S2 p10).

## 4. Discussion

In a systematic analysis, we found that the overall age-standardized incidence rate of nutritional deficiency in China remained stable, while the overall age-standardized DALY rate decreased over the 20-year period between 1990 and 2019. The nutritional deficiency subcategories with the greatest burdens in 2019 were vitamin A deficiency, protein-energy malnutrition, and dietary iron deficiency. These problems were mainly concentrated in children below the age of five and adults over the age of 70, as well as in developed provinces such as Zhejiang, Beijing, and Guangdong and rural provinces such as Tibet, Xinjiang, and Hainan. Future studies on nutritional deficiency in China should focus on Zhejiang and Liaoning. The populations in these provinces showed large increases in the rates of nutritional deficiency over the 20-year period, which culminated in high disease burdens in 2019.

Although the overall incidence of nutritional deficiencies in China did not change significantly over the 20-year period, the age-standardized rate of DALYs from nutritional deficiencies decreased. This was likely due to economic improvements, national mandatory legislation, improvements in health services, and more effective monitoring of the nutrition of China’s population. China’s New National Nutritional Program (2017–2030) [3], released in the context of building a healthy population, reinforces existing nutrition programs, emphasizes nationwide actions and programs that target vulnerable populations with disproportionate burdens, and proposes new interventions for people who are older, ill, or living in poor areas.

The age-standardized rate of DALYs from protein-energy malnutrition has decreased significantly over the past 20 years. Nonetheless, protein-energy malnutrition still contributes substantially to the burden of malnutrition in China [7]. In older adults, protein-energy malnutrition is mainly associated with chronic disease, where it results from the malabsorption and loss of nutrients [8]. While mild levels of chronic protein-energy malnutrition have typically been ignored in China’s healthcare, it is necessary to pay more attention to the supply of high-quality food and protein to China’s population in future. Specifically, studies should investigate the protein and energy requirements of different populations under different conditions, publicize nutritional information, encourage breastfeeding, and inform the public on the appropriate weaning products for infants and young children. Older adults, especially those with chronic diseases, should be provided with nutritional supplements. The early detection and treatment of malnutrition through nutritional investigation will be important for realizing the above recommendations.

Dietary iron deficiency was associated with the highest age-standardized rate of DALYs among the various subcategories of nutritional deficiencies considered. In fact, the incidence of this deficiency may have been underestimated due to a shortage of data on this condition. The dietary intake of iron-containing foods or products is considered a safer solution to iron deficiency than oral iron supplements [9]. Studies have shown that an adequate intake of iron from breastmilk can have profound effects on infant brain development in early life [10]. Dietary iron deficiency may result in anemia [11]—the most common type of iron deficiency in China. Although it is questionable whether the traditional plant-based diet of the Chinese people causes iron deficiency [12], iron fortification remains an important aspect of general health [13]. Future research should systematically analyze the rates of iron absorption for various types and combinations of foods commonly consumed by Chinese people, as different dietary patterns are known to affect the bioavailability of iron [14]. Targeted dietary measures instead of dietary approaches that focus only on a single nutrient or food should be used to address the iron deficiency in China’s population [15].

Compared with protein-energy malnutrition and dietary iron deficiency, vitamin A deficiency was mainly concentrated among adolescents and children in China, with the highest age-standardized incidence rate of all nutritional deficiencies in these age groups in 2019. A previous study found that vitamin A levels were positively correlated with levels of family and regional affluence [16]; another study documented especially low levels of vitamin A in children from rural areas [17]. The number of children with a marginal vitamin A deficiency had also increased [18]. In the future, efforts should be made to improve the monitoring of the vitamin A status of key populations, and to clarify the critical points of clinical diagnosis and nutritional intervention for vitamin A deficiency in different age groups, especially infants and young children [19,20].

Geographically, higher age-standardized incidence rates of nutritional deficiencies were concentrated in Zhejiang, Beijing, Guangdong, and other provinces with higher socioeconomic levels. This may have been related to the relatively comprehensive nutrition detection systems established in these provinces, which resulted in the higher detection rates of undernourishment. In addition, nutritional deficiencies in these provinces may have been more attributable to unbalanced nutrient intake than to deprivation. This was reflected in the prevalence of a high oil and salt intake, a rising consumption of sugar-sweetened beverages, and an insufficient intake of whole grains, dark vegetables, milk, and soy among populations in these provinces. Furthermore, nutritional deficiencies in these provinces may have been associated with low rates of exclusive breastfeeding, a preference for yogurt and milk powder, and the early exposure of children to snacks [21,22]. Sound medical service systems, strong health awareness, and frequent tests of nutritional statuses may also have led to a higher detection rate of nutritional deficiencies in these provinces [23].

Higher age-standardized rates of DALYs from nutritional deficiencies were found in Tibet, Xinjiang, Hainan, and other provinces with lower socioeconomic levels. It is likely that other diseases caused by undernutrition contributed to the social and medical burden in these provinces. Owing to the high-altitude location, outdated production technologies, and special customs, Tibetan families lack food resources and mainly rely on tsampa, beef, mutton, and dairy products for nourishment. Consequently, their children have a simple dietary structure. Furthermore, the low level of education and nutrition awareness implies that complementary foods are likely introduced to children’s diets too late in their development. Important measures to tackle Tibetan children’s nutritional deficiencies thus include increasing variety in the food supply and improving the education levels of mothers.

As nutritional deficiencies are one of the main forms of malnutrition, their prevention and control are crucial for improving health on a national scale. (1) Given that the current nutritional deficiencies in various groups of Chinese residents and the research that has been performed on the content of nutrient supplements, there is a need for a scientific evaluation of the use of nutrient supplements by groups or individuals to reveal how supplements should be used. (2) Special attention should be given to the rationale of nutrient supplement use by people in special occupations or special environments. (3) Moreover, guidance on the use of nutrient supplements by residents should be strengthened, and it must be emphasized that nutrient supplements cannot replace meals. Recommendations should be made for increased consumption of calcium-rich foods, such as dairy products, aquatic products, and soy products; and fruits and fresh vegetables rich in vitamin C, especially dark vegetables; increased intake of B vitamins; and moderate consumption of whole grains. Furthermore, a variety of foods should be promoted to ensure the intake of various micronutrients and minerals.

After 2019, the pattern of nutritional deficiency burdens in China may have changed significantly due to the coronavirus disease 2019 (COVID-19) pandemic, as physical isolation policies may have prevented people from maintaining a high-quality and varied diet. There are three major effects of physical isolation policies on the nutritional status of the population. First, provincial pandemic prevention policies may obstruct the transportation of grains, vegetables, and fruits, resulting in a disrupted supply chain in provinces that are highly dependent on the supply of food from other provinces. Consequently, staying at home for a long period requires the storage of large amounts of non-fresh materials. In addition, people may emotionally overeat in response to stress, negative experiences of self-isolation, and/or feelings of boredom [24,25,26]. Having a balanced and healthy dietary routine may help boost the immune system, and thus it is critical to consider the effect of nutritional habits on susceptibility to COVID-19 and its possible long-term complications [27]. This information may inform the development of an important health-system alert for COVID-19 control and for use in other public health emergencies.

To the best of our knowledge, this is the first study to comprehensively examine the burden of nutritional deficiencies across ages and deficiency subcategories at the national and provincial levels in China from 1990 to 2019. The estimates in GBD 2019 are comparable at the global and provincial levels owing to China’s unified and standardized approach to data collection.

Several limitations should be noted. This study is, by nature, an analysis of secondary data from GBD 2019. As for information on other diseases listed in the GBD study, the accuracy of our data for nutritional deficiencies largely depends on the quality and quantity of the data included in the GBD models. This implies that the incidences of nutritional deficiencies in poorer areas of China may have been underestimated due to the lack of unified assessment tools. To achieve an accurate assessment of nutritional status in China, the following challenges must be addressed: the lack of a consistent and universal agreement on the definition and assessment of nutritional deficiencies [28]; the lack of standardization in the tools used for assessing the nutritional statuses of different populations [29]; and the lack of consensus on the best biomarkers and cutoff values [28]. Therefore, a gold standard for diagnosis is needed to facilitate an accurate estimation of the overall incidence of nutritional deficiencies and their prevalence across different settings.

## 5. Conclusions

In conclusion, our study showed that the incidence of overall nutritional deficiency remained stable in China between 1990 and 2019, while the incidence of iodine deficiency showed a slight increase. The resulting DALYs from overall nutritional deficiency, as well as from different subcategories of nutritional deficiency, showed an extensive decline over the 20-year study period. Although the burden of nutritional deficiencies declined in children below the age of five, the burden remained heaviest in this group compared with the other age groups. At the provincial level, a high incidence of nutritional deficiency was reported in provinces with high socioeconomic levels, such Zhejiang, Beijing, and Guangdong; whereas a high rate of DALYs from nutritional deficiency was concentrated in geographically distinct provinces with high levels of poverty, such as Tibet and Xinjiang. In view of the obvious nutritional improvements achieved by food fortification projects in other countries, it is possible that the fortification of food with specific nutrients is more suitable for improving public health in China than encouraging people to increase their intake of healthy foods. To comprehensively and scientifically evaluate the micronutrient intake and nutritional health status of the Chinese population, further research must examine dietary micronutrient requirements, continue to improve the database of dietary and nutrient supplements and fortified food nutrients, and conduct in-depth development of new micronutrient formulations.

## Figures and Tables

**Table 1:** The number, age-standardized rates of incidence, and DALYs and their estimated annual percentage changes (EAPC) from 1990 to 2019 across ages and age groups for nutritional deficiencies and its main subcategories in China. DALYs = disability-adjusted life-years.

	Incidence				DALYs			
	Case (Thousands)	Percentage Change in Absolute Numbers, 1990–2019	Age-Standardized Incidence Rates (per 100,000)	EAPC 1990–2019	Case (Thousands)	Percentage Change in Absolute Numbers, 1990–2019	Age-Standardized DALYs Rates (per 100,000)	EAPC 1990–2019
**Overall**								
Overall nutritional deficiencies	28,569.88 (22,553.25, 35,422.13)	47.90%	2058.90 (1633.50, 2570.63)	0.11 (−0.19, 0.41)	2606.93 (1815.57, 3659.29)	−62.80%	174.78 (122.20, 241.15)	−5.54 (−5.96, −5.12)
Protein-energy malnutrition	27,834.27 (21,809.61, 34,701.83)	50.29%	1996.51(1570.05,2498.19)	0.08(−0.23,0.40)	790.44(551.66,1082.06)	−73.85%	54.57(39.44,74.30)	−6.93 (−7.65,−6.20)
Iodine deficiency	735.61 (574.29, 917.58)	−7.76%	62.40 (48.58, 78.16)	0.63(0.10,1.16)	264.71 (122.77, 521.29)	9.84%	15.57 (7.16, 30.67)	−0.64 (−1.00, −0.28)
Vitamin A deficiency	25,263.60 (21,390.09, 30,021.08)	−79.35%	2113.34 (1764.06, 2553.64)	−5.83 (−6.17, −5.48)	40.32 (25.94, 61.11)	−67.30%	3.69 (2.34, 5.55)	−4.29 (−4.66, −3.93)
Dietary iron deficiency	-	-	-	-	1206.62 (764.18, 1842.70)	−64.05%	80.69 (51.12, 122.73)	−5.88 (−6.16, −5.60)
Other nutritional deficiency	-	-	-	-	304.83 (210.26, 419.73)	14.67%	20.26 (14.30, 27.86)	0.68 (0.00, 1.37)
**Under 5 years**								
Overall nutritional deficiencies	3597.19 (2597.14, 5101.01)	−60.26%	4414.22 (3187.04, 6259.62)	−2.63 (−3.10, −2.16)	182.42 (131.15, 253.72)	−94.31%	223.85 (160.94, 311.35)	−10.53 (−11.00, −10.06)
Protein-energy malnutrition	3587.61 (2589.36, 5091.06)	−60.23%	4402.47 (3177.49, 6247.40)	−2.63 (−3.09, −2.16)	56.01 (44.72, 68.00)	−97.77%	68.74 (54.88, 83.45)	−13.86 (−14.15, −13.56)
Iodine deficiency	9.58 (4.85, 16.76)	−68.81%	11.75 (5.96, 20.56)	−2.83 (−3.71, −1.95)	0.17 (0.06, 0.37)	−82.85%	0.21 (0.08, 0.46)	−5.95 (−7.53, −4.33)
Vitamin A deficiency	3140.90 (1924.50, 4995.49)	−85.35%	3854.30 (2361.61, 6130.12)	−5.10 (−5.28, −4.92)	9.91 (6.08, 15.04)	−81.81%	12.16 (7.46, 18.45)	−5.35 (−5.76, −4.94)
Dietary iron deficiency	-	-	-	-	98.85 (54.38, 158.00)	−81.34%	121.30 (66.74, 193.89)	−5.43 (−5.86, −5.00)
Other nutritional deficiency	-	-	-	-	17.48 (14.36, 21.30)	−83.44%	21.45 (17.62, 26.14)	−2.09 (−3.20, −0.96)
**5–14 years**								
Overall nutritional deficiencies	1848.40 (1234.20, 2677.74)	22.06%	1290.64 (1220.22, 1361.05)	1.12 (0.52, 1.72)	160.13 (99.97, 241.65)	−77.44%	111.66 (90.95, 132.37)	−5.23 (−5.55, −4.90)
Protein-energy malnutrition	1762.30 (1153.92, 2593.48)	34.38%	1230.33 (1161.58, 1299.08)	1.46 (0.78, 2.15)	46.89 (25.91, 78.49)	−45.52%	32.71 (21.50, 43.92)	−3.13 (−3.58, −2.67)
Iodine deficiency	86.10 (61.30, 123.27)	−57.57%	60.31 (45.09, 75.53)	−1.23 (−1.42, −1.03)	3.84 (1.54, 8.02)	−81.55%	2.69 (2.58, 2.82)	−5.28 (−6.63, −3.91)
Vitamin A deficiency	4761.74 (3178.29, 6904.21)	−83.19%	3320.22 (3207.28, 3433.16)	−4.87 (−5.30, −4.44)	8.59 (4.87, 13.70)	−83.13%	5.99 (5.81, 6.17)	−5.83 (−6.13, −5.52)
Dietary iron deficiency	-	-	-	-	88.43 (48.73, 146.05)	−83.52%	61.64 (46.25, 77.03)	−6.04 (−6.37, −5.71)
Other nutritional deficiency	-	-	-	-	12.37 (7.12, 20.08)	−18.80%	8.64(8.42,8.85)	0.46 (−0.15, 1.07)
**15–49 years**								
Overall nutritional deficiencies	12,879.45 (10,024.63, 16,436.56)	103.12%	1759.71 (1677.49, 1841.93)	1.51 (1.13, 1.90)	1147.82 (750.82, 1663.03)	−44.33%	153.02 (128.77, 177.26)	−3.62 (−3.83, −3.42)
Protein-energy malnutrition	12,290.17 (9446.15, 15755.84)	111.84%	1664.20 (1584.24, 1744.16)	1.54 (1.17, 1.90)	306.02 (190.85, 458.77)	37.50%	41.26 (28.67, 53.85)	−0.95 (−1.30, −0.61)
Iodine deficiency	589.28 (443.55, 767.32)	9.29%	95.51 (76.35, 114.66)	1.32 (0.56, 2.09)	168.23 (76.25, 336.26)	−5.02%	22.07 (12.86, 31.28)	−0.40 (−0.78, −0.02)
Vitamin A deficiency	14,682.02 (11,497.90, 18,280.38)	−78.16%	2131.16 (2040.68, 2221.64)	−5.25 (−5.56, −4.93)	14.03 (8.21, 22.83)	−7.32%	2.01 (1.93, 2.10)	−0.57 (−0.96, −0.17)
Dietary iron deficiency	-	-	-	-	522.77 (318.09, 821.77)	−66.27%	68.74 (52.49, 84.99)	−5.98 (−6.23, −5.73)
Other nutritional deficiency	-	-	-	-	136.77 (85.38, 204.73)	40.92%	18.93 (18.66, 19.20)	1.07 (0.37, 1.78)
**50–69 years**								
Overall nutritional deficiencies	7726.77 (5914.47, 9892.05)	310.71%	2095.03 (2005.31, 2184.74)	1.58 (1.35, 1.80)	757.12 (1069.07, 504.77)	2.84%	205.05 (176.98, 233.12)	−3.61 (−3.89, −3.34)
Protein-energy malnutrition	7682.50 (5864.79, 9852.02)	313.23%	2083.01 (1993.55, 2172.46)	1.59 (1.37, 1.82)	209.16 (300.56, 135.50)	117.75%	56.68 (41.92, 71.43)	−1.41 (−1.89, −0.92)
Iodine deficiency	44.27 (24.80, 72.68)	99.82%	12.02 (11.80, 12.24)	−0.82(−0.87,−0.77)	77.00 (151.38, 35.01)	115.52%	20.86 (11.91, 29.82)	−0.27 (−0.67, 0.13)
Vitamin A deficiency	2393.86 (1663.13, 3407.80)	−49.66%	646.79 (596.94, 696.64)	−5.31 (−5.53, −5.09)	6.03 (9.47, 3.60)	177.83%	1.63 (1.55, 1.71)	1.05 (0.79, 1.30)
Dietary iron deficiency	-	-	-	-	377.00 (580.16, 219.71)	−33.54%	102.10 (82.29, 121.90)	−5.59 (−5.84, −5.35)
Other nutritional deficiency	-	-	-	-	87.92 (126.47, 57.01)	151.39%	23.78 (14.22, 33.34)	0.99 (−0.07, 2.07)
**70+ years**								
Overall nutritional deficiencies	2518.07 (1878.90, 3321.93)	375.67%	2328.89 (2234.30, 2423.48)	1.66 (1.46, 1.87)	359.44 (285.47, 450.28)	21.40%	345.89 (309.44, 382.35)	−3.43 (−3.96, −2.90)
Protein-energy malnutrition	2511.69 (1872.02, 3312.45)	376.73%	2323.02 (2228.55, 2417.48)	1.67(1.46,1.88)	172.36(142.93,206.28)	66.34%	170.13(144.57,195.70)	−2.33(−3.11,−1.54)
Iodine deficiency	6.39(3.52,10.49)	153.60%	5.87 (5.62, 6.14)	−0.38 (−0.40, −0.35)	15.48 (7.26, 29.58)	143.82%	14.22 (13.82, 14.64)	−0.54 (−0.92, −0.16)
Vitamin A deficiency	285.08(201.53,399.51)	−54.51%	262.40(230.65,294.15)	−6.09(−6.25,−5.93)	1.75(0.98,2.86)	204.40%	1.61 (1.48, 1.75)	0.90 (0.63, 1.16)
Dietary iron deficiency	-	-	-	-	119.57 (72.20, 185.77)	−30.71%	111.35 (90.67, 132.03)	−6.38 (−6.73, −6.02)
Other nutritional deficiency	-	-	-	-	304.83 (210.26, 304.83)	287.44%	21.43 (14.78, 29.51)	1.86 (1.14, 2.58)

## Data Availability

Data were obtained from the Global Health Data Exchange query tool (http://ghdx.healthdata.org/gbd-results-tool, accessed on 16 July 2022).

## Supplementary Materials

The following supporting information can be downloaded at: https://www.mdpi.com/article/10.3390/nu14193919/s1. Table S1: Age-standardized rates of incidence for nutritional deficiency in 2019, and their estimated annual percentage changes (EAPC) from 1990 to 2019 by province and ages in China; Table S2: Age-standardized rates of DALYs for nutritional deficiency in 2019, and their estimated annual percentage changes (EAPC) from 1990 to 2019 by province and ages in China.
