# Supplementary material for: Burden of Nutritional Deficiencies in China: Findings from the Global Burden of Disease Study 2019
Source: Nutrients. 2022 Sep 21;14(19):3919. doi: 10.3390/nu14193919 (PMC9570758; doi:10.3390/nu14193919)
Supplement: Supplementary file 1 [file nutrients-14-03919-s001.zip › nutrients-1865554-supplementary.pdf]

Burden of Nutritional Deficiencies in China: Findings from the Global Burden of Disease Study 2019

Liyuan Han

Supplementary tables

Supplementary Table S1. Age-standardized rates of incidence for nutritional deficiency in 2019, and their estimated annual percentage changes (EAPC) from 1990 to 2019 by province and ages in China.

	Overall		Under 5 years		5-14 years		15-49 years		50-69 years		70+ years	
	Rate (per 100,000)	EAPC 1990-2019	Rate (per 100,000)	EAPC 1990-2019	Rate (per 100,000)	EAPC 1990-2019	Rate (per 100,000)	EAPC 1990-2019	Rate (per 100,000)	EAPC 1990-2019	Rate (per 100,000)	EAPC 1990-2019
**Anhui**												
Overall nutritional deficiencies	2212.50(1807.90,2706.68)	0.11(-0.19,0.41)	4375.98(3086.48,6052.95)	-2.63(-3.10,-2.16)	1224.05(1155.48,1292.62)	1.12(0.52,1.72)	1883.25(1798.19,1968.31)	1.51(1.13,1.90)	2510.71(2412.50,2608.92)	1.58(1.35,1.80)	2975.17(2868.26,3082.08)	1.66(1.46,1.87)
Protein-energy malnutrition	2111.55(1709.83,2607.89)	0.08(-0.23,0.40)	4358.62(3067.52,6033.91)	-2.63(-3.09,-2.16)	1130.46(1064.56,1196.36)	1.46(0.78,2.15)	1724.37(1642.98,1805.76)	1.54(1.17,1.90)	2498.33(2400.36,2596.29)	1.59(1.37,1.82)	2969.19(2862.39,3075.99)	1.67(1.46,1.88)
Iodine deficiency	100.95(77.22,128.62)	0.63(0.10,1.16)	17.36(8.61,30.22)	-2.83(-3.71,-1.95)	93.59(74.63,112.55)	-1.23(-1.42,-1.03)	158.88(134.17,183.58)	1.32(0.56,2.09)	12.38(11.38,13.46)	-0.82(-0.87,-0.77)	5.98(4.92,7.27)	-0.38(-0.40,-0.35)

Vitamin A deficiency	2680.75(2077.05,3502.13)	-5.83(-6.17,-5.48)	4789.55(2456.94,8650.94)	-5.10(-5.28,-4.92)	4119.49(3993.69,4245.29)	-4.87(-5.30,-4.44)	2734.85(2632.35,2837.35)	-5.25(-5.56,-4.93)	844.74(787.78,901.71)	-5.31(-5.53,-5.09)	343.44(307.12,379.77)	-6.09(-6.25,-5.93)
Dietary iron deficiency	-	-	-	-	-	-	-	-	-	-	-	-
Other nutritional deficiency	-	-	-	-	-	-	-	-	-	-	-	-
**Beijing**												
Overall nutritional deficiencies	3048.59(2350.75,3870.51)	0.91(0.73,1.09)	5759.21(3681.06,8947.78)	-1.20(-1.57,-0.82)	2170.65(2079.33,2261.97)	2.00(1.75,2.26)	2660.91(2559.80,2762.01)	1.88(1.66,2.10)	3254.09(3142.28,3365.90)	1.88(1.70,2.06)	3410.78(3296.32,3525.25)	1.65(1.48,1.82)
Protein-energy malnutrition	3027.22(2333.73,3847.82)	0.93(0.75,1.12)	5753.95(3674.70,8942.57)	-1.19(-1.57,-0.82)	2148.63(2057.77,2239.48)	2.11(1.84,2.38)	2631.26(2530.72,2731.80)	1.95(1.72,2.17)	3243.73(3132.10,3355.36)	1.90(1.71,2.08)	3405.35(3290.97,3519.73)	1.65(1.48,1.83)
Iodine deficiency	21.37(16.28,27.96)	-1.54(-1.67,-1.41)	5.26(2.62,9.41)	-2.93(-3.73,-2.12)	22.02(12.83,31.22)	-2.45(-2.89,-2.00)	29.65(18.98,40.32)	-1.30(-1.57,-1.03)	10.36(8.88,12.09)	-0.78(-0.86,-0.70)	5.43(3.72,7.94)	-0.38(-0.42,-0.34)

Vitamin A deficiency	825.18(623.49,1122.89)	-4.73(-4.83,-4.63)	1439.82(711.58,3047.02)	-3.88(-4.07,-3.69)	1314.17(1243.11,1385.22)	-4.13(-4.28,-3.97)	881.72(823.52,939.92)	-4.33(-4.45,-4.20)	268.38(236.27,300.49)	-4.03(-4.12,-3.94)	111.43(90.74,132.12)	-4.96(-5.28,-4.64)
Dietary iron deficiency	-	-	-	-	-	-	-	-	-	-	-	-
Other nutritional deficiency	-	-	-	-	-	-	-	-	-	-	-	-
**Chongqing**												
Overall nutritional deficiencies	2094.98(1643.38,2637.55)	0.04(-0.25,0.34)	4708.48(3192.95,6963.43)	-1.98(-2.46,-1.49)	1440.24(1365.86,1514.62)	0.97(0.47,1.47)	1728.32(1646.84,1809.80)	0.91(0.52,1.30)	2064.86(1975.79,2153.92)	0.88(0.58,1.17)	2262.31(2169.09,2355.54)	0.85(0.59,1.12)
Protein-energy malnutrition	2061.92(1610.54,2600.24)	0.05(-0.25,0.34)	4701.06(3187.60,6956.40)	-1.98(-2.46,-1.49)	1407.34(1333.81,1480.87)	1.06(0.54,1.58)	1680.30(1599.96,1760.64)	0.93(0.54,1.33)	2052.95(1964.14,2141.75)	0.89(0.59,1.19)	2256.44(2163.33,2349.54)	0.86(0.59,1.12)
Iodine deficiency	33.06(25.74,42.39)	-0.17(-0.53,0.19)	7.42(3.62,12.93)	-2.07(-2.52,-1.61)	32.90(21.66,44.14)	-1.34(-1.65,-1.04)	48.02(34.44,61.60)	0.28(-0.22,0.77)	11.91(10.43,13.60)	-0.38(-0.49,-0.27)	5.88(4.41,7.83)	-0.16(-0.22,-0.11)

Vitamin A deficiency	2165.46(1640.85,2798.31)	-5.72(-6.10,-5.33)	3870.28(1876.16,7164.92)	-5.06(-5.29,-4.82)	3407.47(3293.06,3521.88)	-4.88(-5.36,-4.41)	2254.59(2161.52,2347.65)	-5.23(-5.61,-4.86)	688.15(636.74,739.57)	-5.16(-5.47,-4.84)	278.03(245.34,310.71)	-6.05(-6.28,-5.83)
Dietary iron deficiency	-	-	-	-	-	-	-	-	-	-	-	-
Other nutritional deficiency	-	-	-	-	-	-	-	-	-	-	-	-
**Fujian**												
Overall nutritional deficiencies	2135.28(1712.84,2629.57)	0.11(-0.18,0.41)	4229.99(2970.57,5948.04)	-2.32(-2.63,-2.00)	1191.10(1123.46,1258.75)	1.15(0.56,1.75)	1795.66(1712.60,1878.71)	1.38(1.01,1.74)	2460.70(2363.48,2557.93)	1.39(1.17,1.62)	2889.52(2784.16,2994.88)	1.43(1.24,1.62)
Protein-energy malnutrition	2086.81(1665.42,2575.54)	0.11(-0.19,0.41)	4219.70(2964.05,5941.24)	-2.32(-2.63,-2.00)	1142.75(1076.49,1209.00)	1.35(0.70,2.00)	1723.42(1642.05,1804.79)	1.40(1.04,1.76)	2448.08(2351.11,2545.06)	1.41(1.18,1.63)	2883.47(2778.22,2988.71)	1.43(1.24,1.62)
Iodine deficiency	48.47(37.71,61.49)	0.27(-0.21,0.75)	10.28(5.26,17.35)	-2.65(-3.48,-1.81)	48.35(34.73,61.98)	-1.40(-1.61,-1.18)	72.24(55.58,88.90)	0.94(0.22,1.67)	12.62(11.31,14.08)	-0.48(-0.56,-0.40)	6.06(4.55,8.07)	-0.20(-0.24,-0.17)

Vitamin A deficiency	1917.66(1462.19,2501.62)	-5.51(-5.73,-5.29)	3304.29(1572.95,6422.82)	-4.86(-4.99,-4.74)	2904.90(2799.26,3010.53)	-4.64(-4.94,-4.34)	1941.05(1854.70,2027.40)	-4.91(-5.12,-4.69)	594.28(546.50,642.06)	-4.84(-4.99,-4.69)	242.97(212.41,273.52)	-5.69(-5.91,-5.48)
Dietary iron deficiency	-	-	-	-	-	-	-	-	-	-	-	-
Other nutritional deficiency	-	-	-	-	-	-	-	-	-	-	-	-
**Gansu**												
Overall nutritional deficiencies	2163.59(1758.56,2672.23)	0.05(-0.28,0.37)	4360.45(3178.67,6111.51)	-2.15(-2.50,-1.80)	1446.76(1372.21,1521.31)	0.90(0.35,1.45)	1923.03(1837.08,2008.98)	1.25(0.78,1.72)	2106.88(2016.92,2196.85)	1.08(0.78,1.38)	2348.31(2253.33,2443.29)	0.98(0.73,1.23)
Protein-energy malnutrition	1985.66(1586.83,2488.96)	-0.03(-0.36,0.31)	4334.36(3154.01,6079.33)	-2.15(-2.49,-1.80)	1287.28(1216.96,1357.60)	1.37(0.71,2.04)	1636.43(1557.14,1715.72)	1.20(0.75,1.65)	2095.36(2005.64,2185.07)	1.10(0.79,1.40)	2342.60(2247.74,2437.47)	0.98(0.73,1.24)
Iodine deficiency	177.93(138.47,226.08)	0.75(0.20,1.30)	26.09(12.95,44.68)	-3.34(-4.07,-2.60)	159.48(134.73,184.23)	-1.25(-1.40,-1.10)	286.60(253.42,319.78)	1.46(0.70,2.23)	11.53(10.06,13.21)	-1.01(-1.07,-0.95)	5.71(4.92,6.63)	-0.51(-0.56,-0.47)

Vitamin A deficiency	3444.42(2641.30,4503.63)	-5.55(-5.88,-5.22)	6142.94(2857.91,11117.01)	-4.76(-4.92,-4.60)	5148.63(5007.99,5289.27)	-4.65(-5.03,-4.27)	3466.38(3350.98,3581.78)	-5.03(-5.32,-4.74)	1069.23(1005.14,1133.32)	-5.10(-5.29,-4.92)	434.21(393.36,475.05)	-5.90(-6.05,-5.75)
Dietary iron deficiency	-	-	-	-	-	-	-	-	-	-	-	-
Other nutritional deficiency	-	-	-	-	-	-	-	-	-	-	-	-
**Guangdong**												
Overall nutritional deficiencies	2892.10(2294.95,3583.66)	1.07(0.86,1.28)	5097.45(3410.94,7644.97)	-1.63(-1.92,-1.35)	1625.47(1546.45,1704.49)	2.46(2.10,2.81)	2535.80(2437.10,2634.50)	2.38(2.28,2.48)	3539.54(3422.93,3656.15)	2.24(1.93,2.55)	4058.02(3933.16,4182.87)	2.26(1.85,2.66)
Protein-energy malnutrition	2851.67(2258.60,3541.27)	1.09(0.86,1.32)	5088.54(3404.67,7639.88)	-1.63(-1.91,-1.35)	1584.49(1506.47,1662.51)	2.65(2.26,3.03)	2476.57(2379.03,2574.11)	2.44(2.31,2.57)	3527.06(3410.66,3643.46)	2.26(1.95,2.57)	4052.00(3927.23,4176.76)	2.26(1.86,2.67)
Iodine deficiency	40.43(31.46,51.65)	0.11(-0.36,0.58)	8.91(4.52,15.32)	-2.27(-3.02,-1.52)	40.98(28.44,53.53)	-1.22(-1.45,-1.00)	59.23(44.14,74.31)	0.64(-0.07,1.35)	12.48(11.65,13.37)	-0.37(-0.49,-0.26)	6.02(5.03,7.21)	-0.16(-0.21,-0.11)

Vitamin A deficiency	1505.08(1130.06,1972.61)	-5.36(-5.55,-5.16)	2487.95(1228.05,4750.66)	-4.82(-5.00,-4.64)	2236.89(2144.19,2329.59)	-4.62(-4.85,-4.39)	1489.40(1413.76,1565.04)	-4.90(-5.06,-4.73)	454.74(412.94,496.53)	-4.76(-4.88,-4.64)	185.28(158.60,211.96)	-5.66(-5.92,-5.40)
Dietary iron deficiency	-	-	-	-	-	-	-	-	-	-	-	-
Other nutritional deficiency	-	-	-	-	-	-	-	-	-	-	-	-
**Guangxi**												
Overall nutritional deficiencies	2022.84(1658.68,2451.17)	1.06(0.95,1.17)	5531.57(4368.76,7208.93)	-0.91(-1.06,-0.76)	1080.49(1016.07,1144.92)	2.14(1.62,2.66)	1543.24(1466.24,1620.24)	2.38(2.12,2.64)	2089.18(1999.60,2178.77)	2.55(2.40,2.70)	2322.17(2227.72,2416.62)	2.52(2.36,2.68)
Protein-energy malnutrition	1998.66(1636.71,2427.27)	1.10(0.99,1.21)	5525.77(4363.28,7201.64)	-0.91(-1.06,-0.76)	1055.96(992.27,1119.65)	2.37(1.81,2.94)	1509.18(1433.04,1585.32)	2.50(2.24,2.77)	2078.33(1988.97,2167.68)	2.58(2.42,2.73)	2316.60(2222.26,2410.94)	2.53(2.37,2.69)
Iodine deficiency	24.17(18.63,30.74)	-1.22(-1.41,-1.02)	5.80(2.81,10.02)	-2.95(-3.81,-2.08)	24.53(14.82,34.24)	-2.34(-2.84,-1.83)	34.06(22.62,45.50)	-0.86(-1.20,-0.53)	10.86(9.72,12.13)	-0.62(-0.72,-0.52)	5.57(4.29,7.23)	-0.29(-0.35,-0.24)

Vitamin A deficiency	2728.73(2066.39,3563.33)	-4.98(-5.08,-4.89)	4669.96(2342.73,8770.55)	-5.13(-5.34,-4.91)	3798.29(3677.49,3919.08)	-4.23(-4.44,-4.02)	2616.26(2516.01,2716.51)	-4.47(-4.58,-4.36)	796.73(741.41,852.06)	-4.40(-4.56,-4.25)	322.50(287.30,357.70)	-5.22(-5.54,-4.90)
Dietary iron deficiency	-	-	-	-	-	-	-	-	-	-	-	-
Other nutritional deficiency	-	-	-	-	-	-	-	-	-	-	-	-
**Guizhou**												
Overall nutritional deficiencies	1969.08(1600.35,2396.71)	1.06(0.86,1.26)	4890.94(3629.36,6712.10)	-1.55(-1.94,-1.16)	1128.66(1062.81,1194.51)	2.42(1.99,2.85)	1603.77(1525.28,1682.26)	2.74(2.40,3.08)	1971.34(1884.31,2058.36)	2.80(2.64,2.96)	2277.84(2184.30,2371.39)	2.80(2.66,2.94)
Protein-energy malnutrition	1863.35(1498.61,2282.36)	1.09(0.88,1.30)	4873.24(3615.49,6696.00)	-1.54(-1.93,-1.16)	1031.27(968.33,1094.21)	3.15(2.63,3.68)	1436.83(1362.54,1511.13)	2.95(2.66,3.25)	1958.98(1872.23,2045.73)	2.84(2.67,3.00)	2271.90(2178.48,2365.32)	2.81(2.67,2.96)
Iodine deficiency	105.73(81.14,135.32)	0.68(0.16,1.20)	17.70(8.96,30.94)	-3.22(-3.75,-2.68)	97.39(78.05,116.74)	-1.39(-1.47,-1.30)	166.93(141.61,192.26)	1.44(0.74,2.16)	12.36(10.94,13.97)	-0.84(-0.88,-0.80)	5.94(4.45,7.92)	-0.42(-0.46,-0.38)

Vitamin A deficiency	3736.08(2867.42,4977.80)	-5.64(-5.89,-5.40)	6981.07(3504.72,12566.07)	-5.18(-5.31,-5.04)	5227.40(5085.69,5369.11)	-4.87(-5.19,-4.55)	3542.74(3426.07,3659.40)	-5.23(-5.44,-5.01)	1091.98(1027.21,1156.75)	-5.40(-5.53,-5.27)	443.14(401.88,484.40)	-6.18(-6.35,-6.01)
Dietary iron deficiency	-	-	-	-	-	-	-	-	-	-		-
Other nutritional deficiency	-	-	-	-	-	-	-	-	-	-		-
**Hainan**												
Overall nutritional deficiencies	2188.20(1792.88,2650.05)	-0.58(-0.88,-0.28)	4799.80(3178.95,7021.12)	-2.70(-3.12,-2.28)	1098.38(1033.43,1163.34)	0.10(-0.35,0.56)	1824.76(1741.03,1908.48)	0.86(0.58,1.14)	2438.48(2341.69,2535.26)	0.93(0.71,1.15)	2879.14(2773.97,2984.31)	1.05(0.83,1.27)
Protein-energy malnutrition	2056.89(1659.62,2522.81)	-0.66(-0.99,-0.32)	4778.33(3158.49,6996.39)	-2.70(-3.12,-2.28)	976.76(915.51,1038.02)	0.44(-0.09,0.98)	1616.41(1537.61,1695.21)	0.77(0.49,1.05)	2426.38(2329.84,2522.93)	0.94(0.72,1.17)	2873.31(2768.24,2978.37)	1.05(0.83,1.27)
Iodine deficiency	131.31(101.06,169.32)	0.62(0.07,1.17)	21.47(11.09,38.76)	-3.68(-4.35,-3.01)	121.62(100.01,143.24)	-1.59(-1.68,-1.50)	208.34(180.05,236.63)	1.43(0.68,2.19)	12.09(9.57,15.28)	-0.93(-0.98,-0.88)	5.84(3.10,10.99)	-0.47(-0.50,-0.43)

Vitamin A deficiency	2419.52(1833.68,3260.92)	-4.88(-5.22,-4.55)	4020.45(1960.70,7573.92)	-4.29(-4.49,-4.10)	3558.75(3441.83,3675.68)	-4.08(-4.50,-3.66)	2357.95(2262.77,2453.12)	-4.41(-4.72,-4.09)	717.94(665.43,770.46)	-4.30(-4.56,-4.04)	293.89(260.29,327.49)	-5.20(-5.40,-5.00)
Dietary iron deficiency	-	-	-	-	-	-	-	-	-	-	-	-
Other nutritional deficiency	-	-	-	-	-	-	-	-	-	-	-	-
**Hebei**												
Overall nutritional deficiencies	1860.91(1464.98,2349.67)	0.28(0.12,0.43)	4167.14(2998.27,5863.19)	-1.46(-1.63,-1.29)	1311.09(1240.12,1382.05)	0.98(0.63,1.33)	1553.46(1476.21,1630.71)	1.21(0.97,1.46)	1732.42(1650.84,1814.00)	1.23(1.11,1.36)	1870.41(1785.65,1955.18)	1.10(0.98,1.21)
Protein-energy malnutrition	1798.35(1403.52,2280.49)	0.27(0.11,0.43)	4154.75(2988.38,5847.08)	-1.45(-1.62,-1.28)	1250.28(1180.98,1319.58)	1.17(0.80,1.55)	1458.18(1383.34,1533.03)	1.22(0.99,1.45)	1719.62(1638.34,1800.90)	1.25(1.12,1.38)	1864.32(1779.69,1948.95)	1.10(0.98,1.22)
Iodine deficiency	62.56(48.01,79.82)	0.41(-0.09,0.92)	12.39(6.07,21.26)	-2.84(-3.38,-2.30)	60.81(45.52,76.09)	-1.42(-1.48,-1.36)	95.28(76.15,114.41)	1.13(0.42,1.85)	12.80(11.87,13.81)	-0.58(-0.64,-0.53)	6.09(5.01,7.40)	-0.28(-0.29,-0.26)

Vitamin A deficiency	2184.58(1667.00,2954.19)	-5.38(-5.59,-5.18)	3699.00(1844.73,7060.24)	-4.81(-4.96,-4.66)	3245.89(3134.22,3357.55)	-4.65(-4.95,-4.34)	2189.75(2098.03,2281.47)	-4.96(-5.18,-4.73)	657.38(607.13,707.63)	-4.88(-5.03,-4.74)	274.59(242.11,307.06)	-5.67(-5.89,-5.45)
Dietary iron deficiency	-	-	-	-	-	-	-	-	-	-	-	-
Other nutritional deficiency	-	-	-	-	-	-	-	-	-	-	-	-
**Heilongjiang**												
Overall nutritional deficiencies	1535.29(1204.27,1965.71)	0.78(0.57,1.00)	3826.15(3086.95,4978.40)	-0.75(-0.92,-0.58)	1053.26(989.65,1116.87)	1.11(0.61,1.61)	1274.70(1204.72,1344.68)	1.65(1.24,2.06)	1333.21(1261.64,1404.78)	1.78(1.62,1.95)	1360.19(1287.90,1432.48)	1.66(1.49,1.84)
Protein-energy malnutrition	1437.16(1110.01,1860.21)	0.80(0.60,0.99)	3809.10(3076.76,4962.78)	-0.74(-0.91,-0.57)	961.16(900.39,1021.92)	1.48(0.89,2.08)	1120.85(1055.23,1186.46)	1.71(1.35,2.06)	1320.84(1249.60,1392.07)	1.82(1.65,1.99)	1354.23(1282.10,1426.35)	1.68(1.50,1.85)
Iodine deficiency	98.12(75.17,126.31)	0.61(0.08,1.14)	17.04(8.61,29.58)	-2.83(-3.57,-2.07)	92.11(73.30,110.92)	-1.20(-1.35,-1.06)	153.86(129.55,178.17)	1.30(0.55,2.06)	12.37(11.23,13.63)	-0.83(-0.87,-0.79)	5.96(4.56,7.81)	-0.40(-0.43,-0.37)

Vitamin A deficiency	1821.73(1406.62,2346.50)	-5.26(-5.33,-5.18)	3448.77(1611.76,6692.13)	-4.44(-4.76,-4.12)	3149.19(3039.20,3259.18)	-4.01(-4.09,-3.93)	2088.46(1998.89,2178.03)	-4.30(-4.39,-4.21)	632.30(583.02,681.59)	-4.15(-4.33,-3.96)	259.02(227.47,290.56)	-4.96(-5.35,-4.58)
Dietary iron deficiency	-	-	-	-	-	-	-	-	-	-	-	-
Other nutritional deficiency	-	-	-	-	-	-	-	-	-	-	-	-
**Henan**												
Overall nutritional deficiencies	1495.20(1130.47,1946.56)	0.78(0.67,0.90)	3671.85(2671.01,5272.85)	-1.12(-1.44,-0.81)	1093.82(1029.00,1158.64)	1.69(1.28,2.09)	1209.74(1141.57,1277.91)	1.74(1.51,1.98)	1335.15(1263.53,1406.76)	1.86(1.72,1.99)	1402.06(1328.67,1475.45)	1.87(1.72,2.01)
Protein-energy malnutrition	1473.85(1112.62,1926.34)	0.85(0.73,0.96)	3666.49(2668.41,5267.22)	-1.12(-1.43,-0.81)	1072.01(1007.83,1136.18)	1.90(1.46,2.34)	1180.07(1112.74,1247.40)	1.90(1.65,2.14)	1324.71(1253.37,1396.05)	1.89(1.75,2.02)	1396.62(1323.37,1469.86)	1.88(1.73,2.02)
Iodine deficiency	21.35(15.88,28.74)	-1.92(-2.03,-1.81)	5.36(2.54,9.66)	-3.25(-3.93,-2.56)	21.81(12.66,30.97)	-2.92(-3.36,-2.47)	29.67(19.00,40.35)	-1.66(-1.87,-1.45)	10.44(9.66,11.27)	-0.90(-0.95,-0.85)	5.44(4.54,6.52)	-0.46(-0.50,-0.43)

Vitamin A deficiency	2418.28(1870.84,3178.85)	-5.51(-5.65,-5.37)	4035.37(2005.76,7432.95)	-5.20(-5.43,-4.97)	3462.65(3347.31,3577.98)	-4.86(-5.02,-4.69)	2332.18(2237.53,2426.83)	-5.11(-5.23,-4.98)	718.64(666.10,771.18)	-5.05(-5.25,-4.85)	291.00(257.56,324.43)	-5.89(-6.27,-5.51)
Dietary iron deficiency	-	-	-	-	-	-	-	-	-	-	-	-
Other nutritional deficiency	-	-	-	-	-	-	-	-	-	-	-	-
**Hong Kong Special Administrative Region of China**												
Overall nutritional deficiencies	1988.87(1521.52,2526.59)	-0.26(-0.62,0.10)	4229.19(2637.38,6750.91)	-1.30(-2.15,-0.44)	1533.61(1456.85,1610.36)	1.24(0.72,1.76)	1648.48(1568.90,1728.06)	0.32(-0.08,0.72)	1828.28(1744.47,1912.08)	-0.33(-0.64,-0.02)	1944.49(1858.06,2030.92)	-0.54(-0.81,-0.27)
Protein-energy malnutrition	1963.99(1498.10,2503.53)	-0.24(-0.61,0.13)	4223.35(2629.76,6744.63)	-1.29(-2.15,-0.43)	1508.80(1432.67,1584.93)	1.39(0.85,1.93)	1613.15(1534.43,1691.88)	0.36(-0.05,0.78)	1817.32(1733.76,1900.87)	-0.33(-0.64,-0.01)	1938.89(1852.58,2025.19)	-0.54(-0.81,-0.27)
Iodine deficiency	24.87(19.07,32.20)	-1.46(-1.53,-1.38)	5.84(2.92,10.15)	-3.61(-4.31,-2.89)	24.81(15.05,34.57)	-2.86(-3.23,-2.49)	35.33(23.68,46.98)	-1.02(-1.25,-0.79)	10.96(8.58,14.01)	-0.71(-0.77,-0.65)	5.60(3.21,9.76)	-0.33(-0.37,-0.30)

Vitamin A deficiency	907.39(647.47,1281.06)	-4.49(-4.63,-4.36)	1634.62(670.31,3360.28)	-3.30(-3.45,-3.15)	1592.17(1513.96,1670.37)	-3.54(-3.76,-3.32)	1002.90(940.83,1064.97)	-3.99(-4.12,-3.87)	303.71(269.55,337.87)	-3.91(-4.01,-3.82)	124.50(102.63,146.37)	-4.70(-4.97,-4.42)
Dietary iron deficiency	-	-	-	-	-	-	-	-	-	-	-	-
Other nutritional deficiency	-	-	-	-	-	-	-	-	-	-	-	-
**Hubei**												
Overall nutritional deficiencies	1448.14(1110.33,1872.68)	0.83(0.70,0.97)	3464.66(2680.50,4742.32)	-1.12(-1.40,-0.83)	930.16(870.38,989.93)	1.73(1.24,2.22)	1170.65(1103.58,1237.71)	1.90(1.64,2.16)	1440.96(1366.56,1515.36)	2.06(1.94,2.19)	1600.02(1521.62,1678.42)	2.08(1.91,2.24)
Protein-energy malnutrition	1420.85(1081.27,1841.69)	0.88(0.74,1.02)	3458.27(2674.73,4738.54)	-1.11(-1.39,-0.83)	902.73(843.84,961.62)	1.97(1.44,2.51)	1131.73(1065.80,1197.67)	2.03(1.77,2.29)	1429.55(1355.45,1503.66)	2.09(1.97,2.22)	1594.28(1516.02,1672.53)	2.09(1.92,2.25)
Iodine deficiency	27.29(21.18,34.96)	-0.91(-1.15,-0.68)	6.39(3.24,11.29)	-2.83(-3.68,-1.97)	27.43(17.16,37.69)	-2.15(-2.61,-1.68)	38.91(26.69,51.14)	-0.50(-0.89,-0.10)	11.41(10.44,12.46)	-0.50(-0.60,-0.40)	5.74(4.65,7.09)	-0.24(-0.28,-0.20)

Vitamin A deficiency	1858.24(1428.97,2407.14)	-6.15(-6.25,-6.06)	3561.77(1652.03,6804.30)	-5.56(-5.73,-5.39)	2923.21(2817.24,3029.18)	-5.05(-5.22,-4.87)	1961.63(1874.82,2048.44)	-5.40(-5.49,-5.32)	601.03(552.97,649.08)	-5.31(-5.46,-5.16)	244.14(213.51,274.76)	-6.13(-6.46,-5.81)
Dietary iron deficiency	-	-	-	-	-	-	-	-	-	-	-	-
Other nutritional deficiency	-	-	-	-	-	-	-	-	-	-	-	-
**Hunan**												
Overall nutritional deficiencies	2165.47(1723.08,2689.58)	1.13(1.03,1.23)	5618.71(4197.15,7709.00)	-1.08(-1.34,-0.82)	1392.39(1319.25,1465.52)	2.66(2.22,3.09)	1684.74(1604.29,1765.19)	2.36(2.03,2.68)	2027.93(1939.67,2116.20)	2.15(1.90,2.40)	2231.67(2139.08,2324.26)	1.91(1.70,2.11)
Protein-energy malnutrition	2144.27(1705.36,2660.81)	1.18(1.07,1.28)	5613.38(4192.94,7701.69)	-1.08(-1.34,-0.82)	1370.67(1298.10,1443.23)	2.86(2.39,3.34)	1655.27(1575.53,1735.01)	2.49(2.15,2.83)	2017.60(1929.57,2105.64)	2.18(1.92,2.43)	2226.29(2133.81,2318.77)	1.91(1.71,2.12)
Iodine deficiency	21.21(15.53,28.55)	-1.85(-1.98,-1.73)	5.32(2.55,9.73)	-3.14(-3.81,-2.46)	21.72(12.58,30.85)	-2.84(-3.27,-2.40)	29.47(18.83,40.11)	-1.59(-1.82,-1.37)	10.33(9.45,11.29)	-0.87(-0.94,-0.80)	5.38(4.37,6.62)	-0.45(-0.48,-0.42)

Vitamin A deficiency	2292.77(1726.53,2949.38)	-5.47(-5.55,-5.40)	4316.90(2163.52,8433.67)	-5.03(-5.22,-4.84)	3448.95(3333.84,3564.06)	-4.63(-4.80,-4.45)	2289.26(2195.48,2383.04)	-4.96(-5.04,-4.87)	703.01(651.04,754.97)	-4.90(-5.07,-4.72)	286.52(253.34,319.70)	-5.74(-6.08,-5.39)
Dietary iron deficiency	-	-	-	-	-	-	-	-	-	-	-	-
Other nutritional deficiency	-	-	-	-	-	-	-	-	-	-	-	-
**Inner Mongolia**												
Overall nutritional deficiencies	2085.27(1646.59,2609.01)	-0.03(-0.34,0.28)	3849.89(2547.24,5794.37)	-2.46(-2.84,-2.07)	1459.89(1385.00,1534.78)	0.75(0.25,1.26)	1859.67(1775.15,1944.19)	1.08(0.65,1.51)	2100.20(2010.37,2190.02)	0.96(0.67,1.25)	2287.64(2193.89,2381.38)	0.83(0.56,1.09)
Protein-energy malnutrition	1976.61(1537.33,2496.68)	-0.07(-0.39,0.25)	3831.52(2524.86,5772.17)	-2.45(-2.83,-2.07)	1357.77(1285.55,1429.99)	1.04(0.46,1.62)	1688.78(1608.23,1769.32)	1.04(0.64,1.45)	2087.89(1998.34,2177.45)	0.98(0.69,1.27)	2281.70(2188.08,2375.33)	0.83(0.57,1.10)
Iodine deficiency	108.65(83.86,138.70)	0.57(0.03,1.11)	18.37(9.25,32.55)	-3.33(-4.29,-2.37)	102.12(82.31,121.93)	-1.45(-1.63,-1.27)	170.89(145.27,196.52)	1.34(0.56,2.12)	12.30(10.85,13.95)	-0.85(-0.91,-0.80)	5.93(4.12,8.55)	-0.44(-0.48,-0.41)

Vitamin A deficiency	1617.35(1230.52,2103.01)	-6.97(-7.32,-6.61)	3037.30(1509.20,5385.58)	-6.37(-6.60,-6.14)	2619.46(2519.15,2719.78)	-5.87(-6.30,-5.44)	1755.31(1673.19,1837.42)	-6.13(-6.48,-5.78)	530.81(485.65,575.97)	-6.13(-6.38,-5.88)	217.89(188.95,246.82)	-6.85(-7.07,-6.63)
Dietary iron deficiency	-	-	-	-	-	-	-	-	-	-	-	-
Other nutritional deficiency	-	-	-	-	-	-	-	-	-	-	-	-
**Jiangsu**												
Overall nutritional deficiencies	1571.59(1168.47,2127.98)	1.09(0.93,1.26)	3281.81(2422.13,4875.12)	-1.18(-1.50,-0.85)	995.68(933.83,1057.53)	1.62(1.20,2.04)	1323.99(1252.68,1395.31)	2.12(1.95,2.29)	1677.59(1597.31,1757.87)	2.41(2.27,2.54)	1906.67(1821.09,1992.26)	2.53(2.31,2.74)
Protein-energy malnutrition	1535.44(1131.44,2087.78)	1.13(0.96,1.30)	3273.82(2415.41,4869.97)	-1.17(-1.49,-0.85)	959.70(898.98,1020.42)	1.85(1.39,2.31)	1271.19(1201.31,1341.07)	2.22(2.06,2.38)	1665.30(1585.32,1745.29)	2.44(2.30,2.58)	1900.72(1815.27,1986.17)	2.54(2.32,2.75)
Iodine deficiency	36.15(27.76,47.34)	-0.02(-0.43,0.39)	8.00(4.12,14.24)	-2.41(-2.96,-1.86)	35.98(24.23,47.74)	-1.45(-1.62,-1.29)	52.80(38.56,67.05)	0.53(-0.08,1.13)	12.29(11.44,13.20)	-0.34(-0.44,-0.24)	5.95(5.06,6.99)	-0.14(-0.19,-0.08)

Vitamin A deficiency	1428.12(1090.97,1925.36)	-5.62(-5.78,-5.46)	2518.76(1280.90,4613.26)	-4.84(-4.99,-4.68)	2310.20(2215.99,2404.40)	-4.75(-4.97,-4.54)	1526.61(1450.03,1603.19)	-5.05(-5.20,-4.89)	471.06(428.52,513.60)	-4.84(-4.95,-4.72)	190.62(163.56,217.68)	-5.76(-6.02,-5.49)
Dietary iron deficiency	-	-	-	-	-	-	-	-	-	-	-	-
Other nutritional deficiency	-	-	-	-	-	-	-	-	-	-	-	-
**Jiangxi**												
Overall nutritional deficiencies	2126.08(1722.36,2585.39)	0.26(-0.09,0.60)	4390.01(3145.34,6160.83)	-2.18(-2.54,-1.82)	1327.64(1256.23,1399.06)	1.40(0.74,2.06)	1808.16(1724.82,1891.51)	1.54(1.05,2.03)	2214.40(2122.16,2306.63)	1.43(1.13,1.74)	2522.84(2424.39,2621.29)	1.38(1.13,1.63)
Protein-energy malnutrition	2029.46(1628.90,2483.39)	0.24(-0.11,0.59)	4372.67(3139.24,6147.68)	-2.18(-2.53,-1.82)	1236.42(1167.50,1305.34)	1.79(1.04,2.54)	1657.06(1577.27,1736.84)	1.57(1.09,2.04)	2201.93(2109.96,2293.90)	1.45(1.14,1.77)	2516.83(2418.50,2615.16)	1.39(1.14,1.64)
Iodine deficiency	96.62(74.00,123.45)	0.58(0.04,1.13)	17.34(8.54,29.85)	-2.96(-3.70,-2.20)	91.22(72.50,109.94)	-1.36(-1.47,-1.25)	151.11(127.01,175.20)	1.31(0.54,2.09)	12.47(11.23,13.84)	-0.80(-0.84,-0.76)	6.01(4.62,7.82)	-0.38(-0.41,-0.34)

Vitamin A deficiency	2619.82(1984.13,3424.65)	-5.60(-5.93,-5.27)	4503.40(2242.86,8835.09)	-4.98(-5.16,-4.79)	3750.47(3630.44,3870.51)	-4.92(-5.34,-4.49)	2537.63(2438.90,2636.37)	-5.21(-5.55,-4.88)	773.22(718.72,827.72)	-5.21(-5.47,-4.95)	315.51(280.69,350.32)	-6.10(-6.27,-5.93)
Dietary iron deficiency	-	-	-	-	-	-	-	-	-	-	-	-
Other nutritional deficiency	-	-	-	-	-	-	-	-	-	-	-	-
**Jilin**												
Overall nutritional deficiencies	1977.22(1567.11,2461.02)	-0.14(-0.46,0.19)	4154.39(2975.81,5797.15)	-2.35(-2.67,-2.04)	1228.75(1160.05,1297.45)	1.15(0.52,1.77)	1642.26(1562.83,1721.69)	1.04(0.60,1.48)	2103.17(2013.28,2193.05)	0.89(0.59,1.19)	2392.67(2296.80,2488.54)	0.77(0.52,1.03)
Protein-energy malnutrition	1940.81(1530.22,2423.94)	-0.14(-0.46,0.19)	4146.28(2967.06,5789.86)	-2.35(-2.67,-2.03)	1192.37(1124.69,1260.05)	1.31(0.64,1.98)	1589.08(1510.94,1667.21)	1.06(0.63,1.51)	2090.98(2001.35,2180.60)	0.90(0.60,1.20)	2386.73(2290.97,2482.48)	0.77(0.52,1.03)
Iodine deficiency	36.40(28.11,46.86)	-0.12(-0.48,0.25)	8.11(4.19,14.18)	-2.65(-3.46,-1.82)	36.38(24.56,48.21)	-1.64(-1.98,-1.31)	53.18(38.89,67.47)	0.48(-0.10,1.07)	12.19(10.85,13.70)	-0.37(-0.47,-0.27)	5.94(4.33,8.16)	-0.14(-0.19,-0.09)

Vitamin A deficiency	1551.14(1186.23,2022.61)	-6.17(-6.43,-5.90)	2984.15(1401.21,6070.63)	-5.02(-5.16,-4.88)	2625.34(2524.91,2725.76)	-5.07(-5.40,-4.73)	1744.85(1662.98,1826.72)	-5.33(-5.59,-5.07)	535.24(489.89,580.58)	-5.21(-5.38,-5.05)	216.35(187.52,245.18)	-6.06(-6.22,-5.89)
Dietary iron deficiency	-	-	-	-	-	-	-	-	-	-	-	-
Other nutritional deficiency	-	-	-	-	-	-	-	-	-	-	-	-
**Liaoning**												
Overall nutritional deficiencies	2189.27(1744.85,2731.65)	1.24(1.11,1.37)	5276.99(4001.59,7146.74)	-0.70(-0.91,-0.49)	1368.92(1296.40,1441.43)	1.91(1.53,2.28)	1788.19(1705.30,1871.07)	2.28(1.97,2.59)	2158.57(2067.51,2249.63)	2.49(2.29,2.69)	2361.55(2266.30,2456.80)	2.45(2.26,2.64)
Protein-energy malnutrition	2118.37(1678.67,2665.23)	1.27(1.15,1.39)	5263.39(3987.06,7138.71)	-0.69(-0.90,-0.49)	1300.19(1229.52,1370.87)	2.17(1.76,2.58)	1679.44(1599.11,1759.76)	2.39(2.11,2.66)	2145.86(2055.06,2236.65)	2.52(2.31,2.73)	2355.46(2260.33,2450.58)	2.46(2.26,2.66)
Iodine deficiency	70.89(54.73,90.88)	0.48(-0.04,1.00)	13.60(6.88,23.00)	-2.32(-2.79,-1.84)	68.72(52.48,84.97)	-1.02(-1.20,-0.84)	108.75(88.31,129.19)	1.07(0.35,1.78)	12.71(11.64,13.88)	-0.66(-0.71,-0.62)	6.10(4.85,7.65)	-0.30(-0.32,-0.28)

Vitamin A deficiency	1452.09(1105.13,1869.98)	-5.00(-5.08,-4.92)	2716.93(1306.88,5104.33)	-3.91(-4.13,-3.69)	2472.16(2374.71,2569.61)	-4.00(-4.16,-3.85)	1662.33(1582.41,1742.24)	-4.18(-4.25,-4.11)	504.52(460.50,548.55)	-4.01(-4.15,-3.87)	208.33(180.04,236.62)	-4.80(-5.13,-4.47)
Dietary iron deficiency	-	-	-	-	-	-	-	-	-	-	-	-
Other nutritional deficiency	-	-	-	-	-	-	-	-	-	-	-	-
**Macao Special Administrative Region of China**												
Overall nutritional deficiencies	2154.12(1719.83,2680.76)	-0.14(-0.40,0.13)	4805.03(3280.29,7043.09)	-1.09(-1.69,-0.48)	1277.53(1207.48,1347.59)	0.41(-0.05,0.87)	1771.34(1688.85,1853.83)	0.48(0.20,0.77)	2347.13(2252.17,2442.08)	0.52(0.32,0.71)	2763.37(2660.34,2866.40)	0.62(0.46,0.78)
Protein-energy malnutrition	2129.90(1695.99,2654.28)	-0.11(-0.38,0.16)	4799.37(3273.44,7039.90)	-1.08(-1.69,-0.48)	1253.19(1183.81,1322.58)	0.53(0.05,1.02)	1737.11(1655.42,1818.80)	0.54(0.25,0.83)	2336.24(2241.51,2430.98)	0.53(0.33,0.72)	2757.86(2654.93,2860.79)	0.62(0.46,0.78)
Iodine deficiency	24.22(18.86,31.59)	-1.63(-1.71,-1.55)	5.66(2.89,9.94)	-3.70(-4.37,-3.03)	24.34(14.67,34.01)	-2.88(-3.24,-2.52)	34.23(22.76,45.70)	-1.27(-1.50,-1.03)	10.89(4.64,25.53)	-0.71(-0.77,-0.65)	5.51(0.49,61.84)	-0.38(-0.41,-0.34)

Vitamin A deficiency	1059.30(737.67,1484.74)	-4.33(-4.59,-4.08)	1875.99(797.48,3899.12)	-3.51(-3.70,-3.32)	1663.77(1583.82,1743.71)	-3.47(-3.81,-3.12)	1093.12(1028.31,1157.92)	-3.79(-4.03,-3.55)	324.27(288.98,359.57)	-3.77(-3.96,-3.59)	133.04(110.44,155.65)	-4.49(-4.71,-4.27)
Dietary iron deficiency	-	-	-	-	-	-	-	-	-	-	-	-
Other nutritional deficiency	-	-	-	-	-	-	-	-	-	-	-	-
**Ningxia**												
Overall nutritional deficiencies	2009.19(1615.76,2513.41)	0.67(0.51,0.82)	3902.05(2723.10,5662.60)	-1.89(-2.21,-1.57)	1298.88(1228.24,1369.52)	1.56(1.20,1.91)	1749.36(1667.38,1831.34)	1.91(1.64,2.18)	2127.19(2036.79,2217.59)	1.96(1.82,2.10)	2399.52(2303.51,2495.53)	1.85(1.71,2.00)
Protein-energy malnutrition	1920.01(1520.74,2422.65)	0.68(0.51,0.84)	3886.17(2706.19,5650.86)	-1.89(-2.21,-1.57)	1214.45(1146.15,1282.76)	1.95(1.54,2.37)	1610.36(1531.70,1689.01)	1.97(1.74,2.20)	2114.58(2024.45,2204.71)	1.99(1.84,2.13)	2393.54(2297.64,2489.43)	1.86(1.72,2.00)
Iodine deficiency	89.18(68.81,113.91)	0.55(0.04,1.07)	15.89(7.89,27.60)	-3.33(-4.08,-2.57)	84.42(66.41,102.43)	-1.54(-1.65,-1.43)	139.00(115.89,162.11)	1.36(0.62,2.11)	12.62(9.59,16.59)	-0.75(-0.78,-0.71)	5.99(2.79,12.85)	-0.36(-0.38,-0.34)

Vitamin A deficiency	2416.99(1791.96,3159.85)	-6.05(-6.45,-5.66)	4059.34(1893.43,7864.37)	-5.75(-5.97,-5.53)	3554.50(3437.64,3671.35)	-5.18(-5.65,-4.71)	2338.46(2243.68,2433.25)	-5.55(-5.92,-5.17)	717.78(665.26,770.29)	-5.53(-5.81,-5.25)	293.63(260.05,327.22)	-6.34(-6.52,-6.15)
Dietary iron deficiency	-	-	-	-	-	-	-	-	-	-	-	-
Other nutritional deficiency	-	-	-	-	-	-	-	-	-	-	-	-
**Qinghai**												
Overall nutritional deficiencies	1957.57(1571.86,2405.30)	0.13(-0.27,0.52)	4561.53(3353.65,6293.29)	-1.90(-2.31,-1.48)	1253.52(1184.13,1322.92)	1.18(0.47,1.90)	1619.59(1540.72,1698.47)	1.27(0.73,1.81)	1945.41(1858.96,2031.86)	1.16(0.79,1.52)	2131.56(2041.07,2222.05)	1.05(0.73,1.37)
Protein-energy malnutrition	1892.99(1511.17,2339.39)	0.11(-0.29,0.51)	4548.94(3337.94,6276.14)	-1.89(-2.31,-1.48)	1190.41(1122.78,1258.03)	1.41(0.63,2.19)	1521.20(1444.76,1597.65)	1.28(0.74,1.83)	1932.64(1846.48,2018.81)	1.18(0.81,1.55)	2125.46(2035.10,2215.82)	1.06(0.74,1.38)
Iodine deficiency	64.57(50.20,81.87)	0.46(-0.05,0.97)	12.59(6.28,22.27)	-2.54(-3.13,-1.94)	63.12(47.55,78.69)	-1.18(-1.31,-1.04)	98.39(78.95,117.83)	1.11(0.39,1.84)	12.77(9.61,16.97)	-0.59(-0.64,-0.55)	6.10(2.67,13.92)	-0.28(-0.31,-0.26)

Vitamin A deficiency	3073.30(2380.22,3980.90)	-5.36(-5.75,-4.98)	5233.77(2603.72,9937.01)	-4.76(-4.97,-4.55)	4477.10(4345.96,4608.25)	-4.57(-5.02,-4.12)	3019.98(2912.27,3127.69)	-4.89(-5.24,-4.53)	924.63(865.03,984.23)	-4.88(-5.15,-4.61)	373.85(335.96,411.75)	-5.79(-5.95,-5.64)
Dietary iron deficiency	-	-	-	-	-	-	-	-	-	-	-	-
Other nutritional deficiency	-	-	-	-	-	-	-	-	-	-	-	-
**Shaanxi**												
Overall nutritional deficiencies	2067.97(1671.18,2540.29)	-0.24(-0.57,0.08)	4208.94(3034.38,5914.38)	-2.42(-2.85,-1.99)	1291.74(1221.30,1362.19)	0.98(0.39,1.58)	1784.46(1701.67,1867.26)	1.08(0.66,1.50)	2175.96(2084.53,2267.39)	0.88(0.62,1.15)	2461.25(2364.01,2558.49)	0.74(0.52,0.97)
Protein-energy malnutrition	1966.03(1570.91,2429.98)	-0.29(-0.63,0.05)	4191.27(3020.75,5903.93)	-2.42(-2.85,-1.98)	1195.61(1127.84,1263.39)	1.28(0.62,1.94)	1624.65(1545.64,1703.65)	1.05(0.65,1.46)	2163.54(2072.38,2254.71)	0.90(0.63,1.16)	2455.32(2358.20,2552.44)	0.75(0.52,0.98)
Iodine deficiency	101.93(78.28,127.75)	0.59(0.05,1.13)	17.67(8.89,32.03)	-2.88(-3.34,-2.42)	96.13(76.91,115.35)	-1.17(-1.32,-1.02)	159.82(135.04,184.60)	1.24(0.52,1.96)	12.41(11.15,13.82)	-0.84(-0.88,-0.80)	5.93(4.51,7.79)	-0.44(-0.47,-0.41)

Vitamin A deficiency	1903.67(1452.58,2477.22)	-6.60(-6.97,-6.23)	3366.70(1672.75,6294.03)	-5.84(-6.05,-5.64)	2946.56(2840.17,3052.95)	-5.68(-6.12,-5.24)	1965.12(1878.23,2052.00)	-6.02(-6.37,-5.67)	608.34(560.00,656.69)	-5.97(-6.21,-5.72)	245.52(214.80,276.23)	-6.76(-6.92,-6.61)
Dietary iron deficiency	-	-	-	-	-	-	-	-	-	-	-	-
Other nutritional deficiency	-	-	-	-	-	-	-	-	-	-	-	-
**Shandong**												
Overall nutritional deficiencies	1190.50(836.46,1733.96)	0.25(-0.06,0.55)	2899.30(2041.32,5236.28)	-0.62(-0.80,-0.45)	979.94(918.59,1041.30)	0.48(-0.04,1.00)	1028.21(965.36,1091.06)	0.80(0.36,1.24)	869.26(811.48,927.05)	0.49(0.27,0.72)	820.17(764.04,876.30)	0.33(0.21,0.46)
Protein-energy malnutrition	1093.02(745.29,1638.89)	0.21(-0.09,0.51)	2882.17(2017.41,5215.52)	-0.60(-0.78,-0.42)	887.74(829.34,946.14)	0.83(0.21,1.44)	875.76(817.76,933.76)	0.68(0.27,1.10)	856.73(799.36,914.10)	0.52(0.29,0.75)	814.20(758.28,870.13)	0.34(0.22,0.47)
Iodine deficiency	97.48(75.33,123.48)	0.56(0.01,1.11)	17.13(8.67,30.18)	-3.17(-3.86,-2.47)	92.20(73.38,111.03)	-1.42(-1.50,-1.33)	152.45(128.25,176.65)	1.29(0.54,2.06)	12.53(11.75,13.37)	-0.79(-0.83,-0.75)	5.97(5.11,6.98)	-0.39(-0.41,-0.36)

Vitamin A deficiency	1678.28(1295.99,2202.57)	-5.36(-5.48,-5.24)	2882.30(1451.46,5576.34)	-5.08(-5.35,-4.82)	2591.18(2491.41,2690.95)	-4.50(-4.66,-4.34)	1722.41(1641.07,1803.75)	-4.82(-4.91,-4.72)	528.29(483.24,573.34)	-4.69(-4.83,-4.56)	213.87(185.21,242.54)	-5.49(-5.83,-5.16)
Dietary iron deficiency	-	-	-	-	-	-	-	-	-	-	-	-
Other nutritional deficiency	-	-	-	-	-	-	-	-	-	-	-	-
**Shanghai**												
Overall nutritional deficiencies	1914.34(1480.27,2404.97)	-0.38(-0.64,-0.11)	4512.05(3083.89,6698.10)	-1.18(-1.38,-0.97)	1349.26(1277.26,1421.25)	0.34(-0.03,0.71)	1562.57(1485.09,1640.04)	0.13(-0.18,0.44)	1803.12(1719.90,1886.35)	0.01(-0.25,0.27)	1921.37(1835.45,2007.28)	-0.16(-0.41,0.09)
Protein-energy malnutrition	1892.30(1457.29,2384.60)	-0.36(-0.63,-0.10)	4506.61(3080.76,6693.16)	-1.18(-1.38,-0.97)	1326.55(1255.16,1397.93)	0.40(0.02,0.79)	1531.89(1455.18,1608.60)	0.16(-0.15,0.47)	1792.65(1709.66,1875.64)	0.01(-0.25,0.27)	1915.93(1830.14,2001.72)	-0.16(-0.41,0.09)
Iodine deficiency	22.04(16.79,29.09)	-1.20(-1.38,-1.01)	5.44(2.56,9.82)	-2.19(-2.76,-1.61)	22.71(13.37,32.05)	-1.85(-2.18,-1.53)	30.67(19.82,41.53)	-1.03(-1.31,-0.75)	10.47(9.10,12.05)	-0.66(-0.75,-0.57)	5.44(3.93,7.51)	-0.32(-0.36,-0.28)

Vitamin A deficiency	923.04(684.87,1233.03)	-4.14(-4.21,-4.07)	1668.34(810.38,3123.44)	-3.38(-3.60,-3.15)	1514.00(1437.73,1590.26)	-3.59(-3.73,-3.45)	1003.78(941.68,1065.88)	-3.84(-3.94,-3.74)	306.30(272.00,340.60)	-3.59(-3.71,-3.46)	124.97(103.06,146.89)	-4.59(-4.93,-4.26)
Dietary iron deficiency	-	-	-	-	-	-	-	-	-	-	-	-
Other nutritional deficiency	-	-	-	-	-	-	-	-	-	-	-	-
**Shanxi**												
Overall nutritional deficiencies	2063.76(1627.98,2570.45)	-0.12(-0.43,0.18)	4401.30(3119.01,6276.21)	-1.93(-2.32,-1.54)	1380.14(1307.32,1452.95)	0.77(0.23,1.31)	1746.47(1664.56,1828.38)	0.88(0.47,1.30)	2110.49(2020.45,2200.53)	0.84(0.55,1.12)	2317.61(2223.26,2411.97)	0.75(0.51,1.00)
Protein-energy malnutrition	2009.96(1580.09,2515.99)	-0.14(-0.45,0.17)	4390.34(3109.64,6265.92)	-1.93(-2.32,-1.54)	1327.20(1255.80,1398.61)	0.91(0.33,1.48)	1665.44(1585.45,1745.43)	0.87(0.47,1.28)	2097.80(2008.03,2187.58)	0.85(0.56,1.14)	2311.55(2217.32,2405.79)	0.76(0.51,1.01)
Iodine deficiency	53.81(41.51,68.77)	0.37(-0.11,0.84)	10.95(5.47,19.20)	-2.69(-3.49,-1.89)	52.93(38.67,67.19)	-1.32(-1.51,-1.13)	81.03(63.39,98.67)	1.05(0.34,1.76)	12.69(11.38,14.15)	-0.51(-0.58,-0.44)	6.06(4.51,8.14)	-0.24(-0.28,-0.20)

Vitamin A deficiency	2099.51(1597.71,2751.27)	-5.90(-6.20,-5.59)	3672.16(1787.55,7166.55)	-5.16(-5.33,-4.99)	3204.23(3093.28,3315.18)	-5.06(-5.41,-4.71)	2138.26(2047.63,2228.90)	-5.36(-5.64,-5.09)	660.63(610.25,711.00)	-5.26(-5.45,-5.07)	265.13(233.22,297.05)	-6.06(-6.25,-5.87)
Dietary iron deficiency	-	-	-	-	-	-	-	-	-	-	-	-
Other nutritional deficiency	-	-	-	-	-	-	-	-	-	-	-	-
**Sichuan**												
Overall nutritional deficiencies	2360.70(1860.03,2940.71)	0.51(0.36,0.67)	4560.70(3221.40,6521.14)	-2.30(-2.81,-1.80)	1607.88(1529.29,1686.47)	2.01(1.58,2.45)	2046.01(1957.35,2134.66)	1.91(1.62,2.20)	2485.53(2387.81,2583.24)	1.77(1.58,1.95)	2747.18(2644.45,2849.91)	1.61(1.46,1.77)
Protein-energy malnutrition	2295.24(1795.55,2872.24)	0.51(0.35,0.68)	4548.07(3209.87,6509.11)	-2.30(-2.80,-1.80)	1544.76(1467.73,1621.80)	2.28(1.80,2.76)	1945.86(1859.40,2032.32)	1.96(1.68,2.24)	2472.78(2375.32,2570.25)	1.78(1.60,1.97)	2741.09(2638.48,2843.71)	1.62(1.47,1.77)
Iodine deficiency	65.45(50.18,83.21)	0.47(-0.04,0.97)	12.63(6.40,22.78)	-2.79(-3.31,-2.26)	63.12(47.55,78.69)	-1.35(-1.41,-1.29)	100.15(80.53,119.76)	1.17(0.47,1.88)	12.74(11.87,13.69)	-0.62(-0.68,-0.55)	6.08(5.19,7.14)	-0.29(-0.32,-0.25)

Vitamin A deficiency	2595.14(2003.84,3339.28)	-6.20(-6.51,-5.90)	4926.96(2463.09,9183.64)	-6.03(-6.18,-5.89)	4070.82(3945.76,4195.87)	-5.25(-5.61,-4.88)	2704.93(2603.00,2806.87)	-5.57(-5.85,-5.29)	829.62(773.17,886.08)	-5.59(-5.76,-5.41)	332.13(296.41,367.85)	-6.46(-6.61,-6.31)
Dietary iron deficiency	-	-	-	-	-	-	-	-	-	-	-	-
Other nutritional deficiency	-	-	-	-	-	-	-	-	-	-	-	-
**Tianjin**												
Overall nutritional deficiencies	1910.08(1501.32,2406.56)	-0.27(-0.59,0.04)	4385.72(2996.82,6407.08)	-1.13(-1.46,-0.81)	1283.71(1213.48,1353.93)	0.07(-0.39,0.52)	1597.29(1518.96,1675.62)	0.36(-0.02,0.74)	1842.38(1758.25,1926.50)	0.32(0.02,0.62)	1958.92(1872.17,2045.67)	0.18(-0.10,0.47)
Protein-energy malnutrition	1860.27(1451.62,2349.85)	-0.29(-0.61,0.03)	4375.10(2985.21,6398.23)	-1.13(-1.45,-0.80)	1232.97(1164.15,1301.80)	0.15(-0.33,0.64)	1523.40(1446.90,1599.90)	0.33(-0.05,0.71)	1829.55(1745.71,1913.38)	0.33(0.02,0.63)	1952.82(1866.20,2039.43)	0.19(-0.10,0.47)
Iodine deficiency	49.81(38.58,64.30)	0.18(-0.30,0.67)	10.63(5.44,18.77)	-2.70(-3.53,-1.87)	50.73(36.77,64.69)	-1.37(-1.57,-1.17)	73.88(57.04,90.73)	0.82(0.09,1.55)	12.83(10.83,15.20)	-0.46(-0.55,-0.38)	6.11(3.96,9.42)	-0.19(-0.23,-0.16)

Vitamin A deficiency	882.28(648.54,1202.22)	-6.02(-6.21,-5.84)	1539.65(728.09,2940.44)	-5.28(-5.42,-5.14)	1417.25(1343.46,1491.04)	-5.36(-5.62,-5.09)	944.33(884.10,1004.56)	-5.57(-5.79,-5.35)	286.46(253.28,319.63)	-5.35(-5.46,-5.23)	119.56(98.13,140.99)	-6.14(-6.35,-5.93)
Dietary iron deficiency	-	-	-	-	-	-	-	-	-	-	-	-
Other nutritional deficiency	-	-	-	-	-	-	-	-	-	-	-	-
**Tibet**												
Overall nutritional deficiencies	1933.62(1576.31,2322.19)	-0.16(-0.57,0.26)	4830.48(3333.77,6877.99)	-1.76(-2.23,-1.28)	1137.26(1071.16,1203.36)	0.65(0.00,1.30)	1654.35(1574.63,1734.07)	1.12(0.57,1.68)	1729.34(1647.83,1810.85)	0.73(0.31,1.14)	1957.52(1870.80,2044.24)	0.63(0.26,1.00)
Protein-energy malnutrition	1706.83(1360.40,2090.08)	-0.29(-0.75,0.18)	4799.32(3311.80,6851.77)	-1.74(-2.22,-1.26)	932.92(873.06,992.79)	1.50(0.60,2.40)	1287.78(1217.45,1358.12)	1.01(0.42,1.60)	1718.28(1637.03,1799.53)	0.74(0.32,1.17)	1951.97(1865.37,2038.56)	0.63(0.26,1.01)
Iodine deficiency	226.79(178.54,281.64)	0.71(0.16,1.26)	31.16(15.83,54.09)	-3.91(-4.61,-3.20)	204.33(176.32,232.35)	-1.37(-1.49,-1.26)	366.57(329.05,404.10)	1.42(0.70,2.15)	11.06(6.81,17.96)	-1.02(-1.09,-0.96)	5.55(1.38,22.27)	-0.53(-0.58,-0.47)

Vitamin A deficiency	8054.81(6197.81,10273.87)	-4.26(-4.51,-4.01)	13200.38(6897.12,23584.31)	-3.89(-4.02,-3.77)	10627.38(10425.33,10829.44)	-3.71(-4.03,-3.38)	7314.77(7147.14,7482.41)	-4.10(-4.33,-3.86)	2345.06(2250.15,2439.98)	-4.41(-4.55,-4.27)	953.68(893.15,1014.21)	-5.32(-5.47,-5.18)
Dietary iron deficiency	-	-	-	-	-	-	-	-	-	-	-	-
Other nutritional deficiency	-	-	-	-	-	-	-	-	-	-	-	-
**Xinjiang**												
Overall nutritional deficiencies	2059.43(1683.07,2511.05)	-0.19(-0.50,0.11)	4774.13(3082.26,7200.30)	-2.01(-2.43,-1.60)	1137.46(1071.36,1203.56)	0.13(-0.33,0.60)	1773.30(1690.76,1855.83)	1.09(0.67,1.51)	1980.83(1893.60,2068.07)	0.91(0.63,1.18)	2327.29(2232.73,2421.84)	0.88(0.63,1.12)
Protein-energy malnutrition	1818.23(1445.78,2254.74)	-0.33(-0.68,0.02)	4742.39(3063.12,7163.30)	-2.00(-2.41,-1.58)	924.70(865.10,984.30)	0.85(0.20,1.50)	1381.20(1308.36,1454.04)	0.92(0.53,1.32)	1969.82(1882.83,2056.81)	0.93(0.65,1.20)	2321.73(2227.29,2416.17)	0.88(0.64,1.13)
Iodine deficiency	241.19(189.17,304.10)	0.75(0.21,1.29)	31.74(16.43,54.57)	-4.18(-5.15,-3.20)	212.76(184.17,241.35)	-1.55(-1.65,-1.44)	392.10(353.29,430.91)	1.56(0.80,2.33)	11.01(9.42,12.88)	-1.06(-1.12,-1.00)	5.56(3.58,8.64)	-0.58(-0.63,-0.52)

Vitamin A deficiency	2430.13(1835.01,3226.57)	-4.83(-5.12,-4.55)	3997.18(1963.63,7233.15)	-4.24(-4.40,-4.08)	3550.79(3433.99,3667.58)	-4.02(-4.36,-3.67)	2375.91(2280.37,2471.45)	-4.37(-4.64,-4.10)	728.49(675.59,781.39)	-4.14(-4.35,-3.93)	295.28(261.60,328.96)	-4.96(-5.14,-4.78)
Dietary iron deficiency	-	-	-	-	-	-	-	-	-	-	-	-
Other nutritional deficiency	-	-	-	-	-	-	-	-	-	-	-	-
**Yunnan**												
Overall nutritional deficiencies	2148.37(1751.74,2610.85)	-0.08(-0.47,0.32)	4972.61(3556.94,6945.07)	-2.89(-3.37,-2.40)	1105.43(1040.26,1170.60)	1.33(0.69,1.98)	1729.69(1648.18,1811.21)	1.60(1.24,1.96)	2435.35(2338.62,2532.07)	1.68(1.42,1.94)	2857.75(2752.97,2962.53)	1.70(1.44,1.96)
Protein-energy malnutrition	2109.48(1713.48,2570.95)	-0.08(-0.48,0.32)	4964.06(3547.15,6936.37)	-2.89(-3.38,-2.40)	1066.44(1002.43,1130.45)	1.50(0.82,2.18)	1672.66(1592.50,1752.82)	1.65(1.29,2.02)	2423.04(2326.56,2519.52)	1.70(1.43,1.96)	2851.76(2747.09,2956.43)	1.70(1.45,1.96)
Iodine deficiency	38.89(30.17,49.97)	0.08(-0.34,0.49)	8.55(4.28,14.79)	-2.02(-2.46,-1.56)	38.99(26.75,51.23)	-1.15(-1.41,-0.89)	57.03(42.23,71.83)	0.56(-0.01,1.14)	12.30(11.08,13.66)	-0.37(-0.48,-0.26)	5.99(4.57,7.85)	-0.17(-0.21,-0.12)

Vitamin A deficiency	3598.64(2734.22,4678.62)	-5.05(-5.38,-4.71)	6327.46(3009.64,11711.70)	-4.89(-5.04,-4.73)	5188.56(5047.38,5329.74)	-4.21(-4.64,-3.78)	3512.76(3396.59,3628.92)	-4.56(-4.89,-4.24)	1090.18(1025.46,1154.89)	-4.56(-4.83,-4.29)	447.79(406.32,489.27)	-5.41(-5.59,-5.22)
Dietary iron deficiency	-	-	-	-	-	-	-	-	-	-	-	-
Other nutritional deficiency	-	-	-	-	-	-	-	-	-	-	-	-
**Zhejiang**												
Overall nutritional deficiencies	3443.36(2667.87,4357.97)	1.21(1.08,1.35)	6027.12(3633.98,9521.97)	-1.67(-2.21,-1.13)	2312.04(2217.80,2406.29)	2.27(1.94,2.61)	3029.80(2921.91,3137.68)	2.31(2.09,2.52)	3844.13(3722.61,3965.66)	2.25(2.06,2.44)	4244.76(4117.06,4372.46)	2.33(2.16,2.49)
Protein-energy malnutrition	3407.36(2626.89,4317.13)	1.23(1.09,1.38)	6019.09(3631.33,9516.01)	-1.67(-2.21,-1.13)	2275.75(2182.25,2369.25)	2.39(2.03,2.75)	2977.38(2870.43,3084.33)	2.36(2.14,2.57)	3831.94(3710.61,3953.27)	2.26(2.07,2.46)	4238.80(4111.19,4366.40)	2.33(2.17,2.50)
Iodine deficiency	36.01(27.94,47.07)	-0.08(-0.48,0.31)	8.03(4.08,14.05)	-2.26(-2.88,-1.63)	36.30(24.49,48.10)	-1.37(-1.67,-1.08)	52.42(38.23,66.61)	0.42(-0.16,1.01)	12.19(11.20,13.28)	-0.36(-0.47,-0.26)	5.96(4.82,7.37)	-0.16(-0.20,-0.11)

Vitamin A deficiency	1579.00(1200.50,2040.03)	-5.81(-6.01,-5.61)	2786.94(1403.39,5401.99)	-5.31(-5.47,-5.14)	2532.05(2433.42,2630.67)	-4.96(-5.23,-4.70)	1677.82(1597.53,1758.10)	-5.24(-5.45,-5.02)	509.51(465.27,553.76)	-5.13(-5.27,-5.00)	210.55(182.11,239.00)	-6.00(-6.23,-5.77)
Dietary iron deficiency	-	-	-	-	-	-	-	-	-	-	-	-
Other nutritional deficiency	-	-	-	-	-	-	-	-	-	-	-	-

Table S2 Age-standardized rates of DALYs for nutritional deficiency in 2019, and their estimated annual percentage changes (EAPC) from 1990 to 2019 by province and ages in China.

	Overall		Under 5 years		5-14 years		15-49 years		50-69 years		70+ years	
	Rate (per 100,000)	EAPC 1990-2019	Rate (per 100,000)	EAPC 1990-2019	Rate (per 100,000)	EAPC 1990-2019	Rate (per 100,000)	EAPC 1990-2019	Rate (per 100,000)	EAPC 1990-2019	Rate (per 100,000)	EAPC 1990-2019
**Anhui**												
Overall nutritional deficiencies	193.52(135.05,269.67)	-5.54(-5.96,-5.12)	205.88(131.06,326.47)	-10.53(-11.00,-10.06)	106.67(86.43,126.92)	-5.23(-5.55,-4.90)	164.76(139.60,189.91)	-3.62(-3.83,-3.42)	230.32(200.57,260.06)	-3.61(-3.89,-3.34)	500.22(456.38,544.05)	-3.43(-3.96,-2.90)
Protein-energy malnutrition	68.29(51.90,89.56)	-6.93(-7.65,-6.20)	60.28(42.80,83.26)	-13.86(-14.15,-13.56)	30.45(19.64,41.27)	-3.13(-3.58,-2.67)	43.02(30.16,55.87)	-0.95(-1.30,-0.61)	69.58(53.23,85.93)	-1.41(-1.89,-0.92)	328.22(292.71,363.73)	-2.33(-3.11,-1.54)
Iodine deficiency	26.66(12.20,52.88)	-0.64(-1.00,-0.28)	0.31(0.08,0.72)	-5.95(-7.53,-4.33)	4.07(3.43,4.83)	-5.28(-6.63,-3.91)	38.09(25.99,50.18)	-0.40(-0.78,-0.02)	35.43(23.76,47.09)	-0.27(-0.67,0.13)	24.44(14.75,34.13)	-0.54(-0.92,-0.16)
Vitamin A deficiency	4.86(3.01,7.62)	-4.29(-4.66,-3.93)	14.98(8.49,23.99)	-5.35(-5.76,-4.94)	7.82(6.91,8.85)	-5.83(-6.13,-5.52)	2.99(2.51,3.58)	-0.57(-0.96,-0.17)	1.91(1.54,2.36)	1.05(0.79,1.30)	1.85(1.30,2.63)	0.90(0.63,1.16)

Dietary iron deficiency	75.72(43.53,117.22)	-5.88(-6.16,-5.60)	119.43(53.37,232.41)	-5.43(-5.86,-5.00)	57.03(42.23,71.83)	-6.04(-6.37,-5.71)	62.41(46.92,77.89)	-5.98(-6.23,-5.73)	97.48(78.13,116.84)	-5.59(-5.84,-5.35)	109.67(89.14,130.19)	-6.38(-6.73,-6.02)
Other nutritional deficiency	17.98(11.88,25.81)	0.68(0.00,1.37)	10.89(6.02,18.82)	-2.09(-3.20,-0.96)	7.31(6.43,8.31)	0.46(-0.15,1.07)	18.25(16.98,19.61)	1.07(0.37,1.78)	25.91(15.94,35.89)	0.99(-0.07,2.07)	36.04(24.28,47.81)	1.86(1.14,2.58)
**Beijing**												
Overall nutritional deficiencies	160.72(102.65,233.65)	-2.51(-2.85,-2.16)	152.63(82.40,273.91)	-5.15(-5.60,-4.68)	131.06(108.62,153.50)	-2.79(-3.18,-2.39)	154.81(130.43,179.20)	-1.44(-1.68,-1.20)	181.54(155.13,207.95)	-1.93(-2.33,-1.52)	246.62(215.84,277.40)	-3.25(-3.70,-2.80)
Protein-energy malnutrition	66.69(41.30,102.79)	-0.60(-0.97,-0.22)	28.70(19.86,39.69)	-8.28(-9.05,-7.50)	57.21(42.38,72.03)	1.77(1.51,2.03)	65.42(49.57,81.28)	1.87(1.65,2.09)	83.11(65.24,100.98)	1.41(1.17,1.65)	116.92(95.73,138.12)	-1.91(-2.38,-1.44)
Iodine deficiency	4.32(1.98,8.31)	-1.60(-1.74,-1.46)	0.09(0.02,0.25)	-2.94(-3.74,-2.13)	1.11(0.55,2.24)	-2.69(-3.36,-2.02)	5.98(5.01,7.15)	-1.62(-1.77,-1.46)	5.67(4.60,6.98)	-1.37(-1.55,-1.18)	4.18(2.71,6.44)	-1.08(-1.23,-0.94)
Vitamin A deficiency	1.53(0.90,2.39)	-2.84(-3.19,-2.48)	4.81(2.47,8.21)	-3.32(-3.66,-2.98)	2.36(1.46,3.82)	-4.16(-4.59,-3.73)	0.75(0.46,1.24)	-1.24(-1.59,-0.88)	1.04(0.64,1.70)	0.01(-0.12,0.14)	1.14(0.50,2.60)	-0.27(-0.36,-0.18)

Dietary iron deficiency	62.53(35.81,101.98)	-4.48(-4.85,-4.10)	114.34(47.55,230.18)	-3.76(-4.08,-3.44)	55.88(41.22,70.53)	-4.98(-5.42,-4.54)	52.64(38.42,66.86)	-4.12(-4.47,-3.78)	57.05(42.24,71.85)	-4.98(-5.42,-4.54)	90.60(71.94,109.26)	-5.28(-5.81,-4.74)
Other nutritional deficiency	25.66(15.67,39.69)	1.22(0.82,1.62)	4.68(2.78,7.84)	-2.01(-3.58,-0.41)	14.51(11.95,17.62)	1.05(0.85,1.25)	30.01(19.28,40.75)	1.52(1.07,1.96)	34.67(23.13,46.21)	1.32(0.35,2.30)	33.79(22.39,45.18)	1.11(0.65,1.59)
**Chongqing**												
Overall nutritional deficiencies	156.06(102.80,227.01)	-4.31(-4.53,-4.09)	186.56(107.69,319.77)	-7.25(-7.77,-6.72)	119.11(97.72,140.50)	-4.34(-4.55,-4.12)	142.61(119.20,166.02)	-3.41(-3.55,-3.26)	186.22(159.48,212.97)	-3.35(-3.50,-3.19)	235.44(205.36,265.51)	-3.88(-4.17,-3.60)
Protein-energy malnutrition	41.68(25.62,63.95)	-4.22(-4.84,-3.59)	29.34(18.83,41.38)	-12.00(-12.97,-11.03)	35.05(23.44,46.65)	0.07(-0.32,0.47)	38.89(26.67,51.11)	0.61(0.28,0.94)	49.72(35.90,63.54)	0.32(0.06,0.57)	76.16(59.05,93.26)	-2.07(-2.57,-1.57)
Iodine deficiency	7.35(3.40,14.46)	0.13(-0.28,0.54)	0.13(0.02,0.35)	-2.05(-2.50,-1.59)	1.60(1.03,2.48)	-1.81(-2.21,-1.40)	10.33(8.88,12.02)	0.17(-0.26,0.60)	9.66(8.34,11.20)	0.43(-0.02,0.88)	6.76(5.17,8.84)	0.48(0.05,0.91)
Vitamin A deficiency	4.26(2.62,6.49)	-4.02(-4.40,-3.64)	16.28(8.86,25.80)	-4.30(-4.75,-3.84)	6.12(4.89,7.66)	-5.86(-6.19,-5.53)	2.18(1.57,3.03)	-1.40(-1.95,-0.84)	1.84(1.31,2.58)	0.59(0.38,0.80)	1.90(1.14,3.15)	0.36(0.23,0.50)

Dietary iron deficiency	84.67(50.17,133.28)	-4.95(-5.14,-4.76)	127.90(55.70,251.66)	-4.39(-4.67,-4.11)	67.23(51.16,83.30)	-5.39(-5.63,-5.15)	73.03(56.28,89.78)	-4.96(-5.14,-4.77)	102.95(83.06,122.84)	-4.70(-4.86,-4.54)	112.20(91.44,132.96)	-5.51(-5.79,-5.23)
Other nutritional deficiency	18.10(11.80,26.62)	-0.97(-1.38,-0.57)	12.90(7.36,22.79)	-6.01(-6.80,-5.21)	9.11(7.58,10.95)	-0.19(-0.58,0.21)	18.18(16.21,20.38)	0.37(-0.25,1.00)	22.06(12.85,31.26)	0.29(-0.67,1.26)	38.42(26.27,50.57)	1.42(0.89,1.95)
**Fujian**												
Overall nutritional deficiencies	155.50(110.31,208.92)	-5.36(-5.73,-4.99)	185.31(121.27,283.36)	-10.11(-10.63,-9.60)	89.68(71.12,108.24)	-4.75(-5.02,-4.49)	124.51(102.64,146.38)	-2.89(-3.03,-2.75)	186.19(159.45,212.94)	-3.11(-3.28,-2.94)	420.19(380.01,460.37)	-3.68(-3.98,-3.39)
Protein-energy malnutrition	63.61(46.73,85.28)	-6.89(-7.59,-6.19)	65.07(44.89,91.06)	-13.10(-13.66,-12.54)	30.94(20.04,41.84)	-2.83(-3.24,-2.41)	43.00(30.14,55.85)	-1.01(-1.31,-0.71)	68.26(52.07,84.45)	-1.72(-2.13,-1.30)	267.13(235.10,299.17)	-3.68(-4.07,-3.28)
Iodine deficiency	11.66(5.41,23.30)	0.60(0.08,1.13)	0.19(0.04,0.48)	-2.63(-3.47,-1.77)	2.26(1.69,3.03)	-2.15(-2.73,-1.57)	16.40(14.99,17.94)	0.67(0.10,1.23)	15.59(14.13,17.21)	0.99(0.39,1.59)	10.91(8.81,13.51)	0.95(0.39,1.53)
Vitamin A deficiency	2.72(1.61,4.17)	-4.15(-4.44,-3.85)	7.61(3.66,12.88)	-5.31(-5.65,-4.96)	4.23(3.42,5.24)	-5.79(-6.09,-5.49)	1.67(1.26,2.21)	-0.94(-1.18,-0.69)	1.57(1.15,2.14)	0.78(0.50,1.05)	1.54(0.87,2.72)	0.74(0.48,1.01)

Dietary iron deficiency	54.96(32.10,86.53)	-5.31(-5.54,-5.08)	89.60(37.62,178.84)	-5.05(-5.37,-4.74)	44.13(31.11,57.15)	-5.83(-6.11,-5.54)	44.07(31.06,57.08)	-5.14(-5.38,-4.90)	72.83(56.10,89.56)	-4.98(-5.17,-4.79)	72.45(55.77,89.13)	-6.16(-6.52,-5.80)
Other nutritional deficiency	22.56(15.94,31.14)	0.41(-0.22,1.04)	22.85(12.55,38.49)	-2.25(-3.27,-1.23)	8.12(6.96,9.47)	0.43(-0.13,1.00)	19.37(17.83,21.04)	0.97(0.30,1.66)	27.94(17.58,38.30)	0.72(-0.22,1.66)	68.16(51.98,84.34)	2.15(1.42,2.90)
**Gansu**												
Overall nutritional deficiencies	212.74(141.88,309.89)	-4.56(-4.74,-4.38)	255.80(163.46,409.64)	-9.27(-9.68,-8.85)	129.33(107.04,151.62)	-4.35(-4.52,-4.17)	202.39(174.50,230.27)	-2.07(-2.30,-1.83)	253.10(221.92,284.28)	-2.41(-2.54,-2.28)	346.30(309.83,382.78)	-3.09(-3.25,-2.92)
Protein-energy malnutrition	48.58(32.73,68.55)	-7.75(-8.49,-7.01)	65.03(42.14,99.48)	-13.29(-13.98,-12.60)	32.36(21.21,43.51)	-1.51(-1.92,-1.10)	38.08(25.99,50.18)	0.10(-0.23,0.43)	53.10(38.82,67.38)	-0.32(-0.63,-0.02)	123.94(102.12,145.76)	-2.75(-3.27,-2.23)
Iodine deficiency	49.13(22.78,96.26)	0.04(-0.36,0.44)	0.47(0.13,1.18)	-6.36(-7.76,-4.94)	6.47(5.25,7.97)	-4.95(-5.99,-3.90)	70.24(53.81,86.67)	0.22(-0.22,0.66)	65.92(50.00,81.83)	0.48(-0.02,0.98)	46.32(32.98,59.66)	0.54(0.06,1.02)
Vitamin A deficiency	4.30(2.64,6.64)	-4.37(-4.56,-4.18)	14.70(8.24,24.87)	-5.11(-5.33,-4.89)	7.30(6.00,8.88)	-5.81(-6.07,-5.55)	2.13(1.57,2.90)	-0.15(-0.29,0.00)	1.83(1.30,2.58)	1.53(1.15,1.91)	1.79(0.90,3.58)	0.98(0.68,1.27)

Dietary iron deficiency	88.96(52.86,135.56)	-4.43(-4.65,-4.22)	140.84(67.95,287.44)	-4.45(-4.64,-4.26)	74.21(57.32,91.09)	-4.98(-5.17,-4.78)	73.89(57.04,90.73)	-4.14(-4.45,-3.81)	108.17(87.79,128.56)	-4.19(-4.40,-3.99)	119.79(98.34,141.24)	-5.05(-5.30,-4.80)
Other nutritional deficiency	21.78(15.53,29.99)	-1.27(-1.90,-0.63)	34.76(18.55,59.96)	-4.53(-5.69,-3.36)	8.99(7.53,10.73)	0.31(-0.23,0.85)	18.04(16.23,20.06)	0.77(0.11,1.43)	24.07(14.46,33.69)	0.72(-0.20,1.64)	54.47(40.00,68.93)	2.38(1.67,3.09)
**Guangdong**												
Overall nutritional deficiencies	196.95(141.77,261.14)	-4.02(-4.49,-3.55)	177.68(113.34,273.13)	-8.43(-8.74,-8.12)	109.68(89.16,130.21)	-3.95(-4.20,-3.70)	158.88(134.18,183.59)	-2.30(-2.48,-2.12)	247.63(216.79,278.48)	-2.49(-2.86,-2.13)	612.79(564.27,661.31)	-3.27(-4.41,-2.11)
Protein-energy malnutrition	91.01(65.90,122.01)	-4.60(-5.58,-3.61)	53.64(39.14,71.36)	-11.53(-11.84,-11.21)	42.91(30.07,55.74)	-1.77(-2.16,-1.39)	64.33(48.61,80.05)	-0.46(-0.93,0.02)	108.74(88.30,129.18)	-1.12(-1.89,-0.35)	417.83(377.76,457.89)	-3.32(-5.01,-1.60)
Iodine deficiency	9.46(4.33,18.66)	0.48(-0.03,1.00)	0.16(0.03,0.40)	-2.32(-3.04,-1.60)	1.96(1.62,2.37)	-1.83(-2.37,-1.28)	13.22(12.53,13.96)	0.51(-0.04,1.07)	12.71(11.88,13.61)	0.86(0.29,1.44)	9.00(7.76,10.42)	0.90(0.34,1.46)
Vitamin A deficiency	2.54(1.51,3.93)	-3.52(-3.89,-3.16)	10.11(5.32,16.46)	-3.84(-4.30,-3.38)	3.82(3.33,4.38)	-4.99(-5.30,-4.67)	1.17(0.97,1.40)	-0.84(-1.21,-0.47)	1.14(0.91,1.43)	0.37(0.18,0.56)	1.01(0.65,1.57)	0.01(-0.13,0.14)

Dietary iron deficiency	62.46(36.43,98.42)	-4.97(-5.21,-4.72)	97.53(40.47,182.99)	-4.77(-5.09,-4.45)	49.82(35.98,63.65)	-5.42(-5.68,-5.15)	51.22(37.19,65.25)	-4.77(-5.10,-4.45)	82.42(64.63,100.22)	-4.69(-4.88,-4.49)	84.33(66.33,102.32)	-5.70(-6.05,-5.35)
Other nutritional deficiency	31.48(21.51,43.60)	1.36(0.84,1.88)	16.24(10.00,25.23)	-1.19(-2.72,0.35)	11.18(10.32,12.11)	1.38(0.97,1.79)	28.94(18.40,39.48)	1.94(1.36,2.51)	42.62(29.82,55.41)	1.43(0.52,2.36)	100.63(80.96,120.29)	1.66(1.01,2.32)
**Guangxi**												
Overall nutritional deficiencies	146.50(105.97,196.21)	-6.33(-6.79,-5.87)	252.84(168.92,364.35)	-9.98(-10.49,-9.47)	88.24(69.82,106.65)	-5.16(-5.47,-4.84)	101.46(81.72,121.20)	-4.36(-4.61,-4.12)	162.24(137.27,187.20)	-4.10(-4.42,-3.77)	412.03(372.24,451.81)	-4.02(-4.65,-3.39)
Protein-energy malnutrition	50.55(39.86,64.01)	-8.17(-8.97,-7.36)	97.88(69.90,136.94)	-12.61(-13.21,-12.01)	18.44(17.08,19.91)	-3.49(-3.97,-3.02)	25.45(15.57,35.34)	-0.87(-1.29,-0.45)	44.80(31.68,57.92)	-1.34(-2.03,-0.65)	241.81(211.33,272.29)	-3.72(-4.63,-2.79)
Iodine deficiency	5.04(2.30,10.00)	-2.32(-3.04,-1.59)	0.10(0.02,0.27)	-4.30(-5.75,-2.82)	1.23(0.91,1.65)	-4.13(-5.52,-2.72)	7.00(6.17,7.95)	-2.24(-2.90,-1.57)	6.66(5.78,7.67)	-2.10(-2.81,-1.38)	4.90(3.71,6.47)	-2.12(-2.96,-1.28)
Vitamin A deficiency	6.47(3.86,10.17)	-3.10(-3.60,-2.60)	21.43(11.80,34.64)	-4.11(-4.73,-3.49)	9.91(8.92,11.00)	-4.41(-4.84,-3.98)	3.96(3.35,4.69)	-0.17(-0.62,0.30)	2.34(1.84,2.97)	0.90(0.67,1.13)	2.23(1.48,3.37)	0.17(-0.08,0.42)

Dietary iron deficiency	67.28(40.42,105.78)	-5.84(-6.13,-5.54)	108.00(47.09,200.52)	-5.28(-5.66,-4.91)	53.43(39.10,67.75)	-5.88(-6.21,-5.55)	53.51(39.18,67.85)	-6.00(-6.30,-5.71)	89.99(71.39,108.58)	-5.54(-5.81,-5.27)	93.16(74.24,112.08)	-6.47(-6.87,-6.07)
Other nutritional deficiency	17.16(13.21,22.28)	0.51(-0.05,1.07)	25.42(15.00,41.06)	-3.36(-4.66,-2.04)	5.24(4.53,6.05)	1.17(0.69,1.65)	11.53(10.44,12.73)	2.20(1.68,2.72)	18.46(16.96,20.09)	2.33(1.50,3.17)	69.93(53.54,86.32)	3.62(2.90,4.35)
**Guizhou**												
Overall nutritional deficiencies	201.06(145.81,275.07)	-7.59(-8.07,-7.10)	311.76(212.14,452.99)	-12.71(-13.32,-12.10)	109.36(88.86,129.86)	-5.32(-5.57,-5.06)	158.37(133.70,183.04)	-3.36(-3.46,-3.26)	226.23(196.75,255.71)	-3.52(-3.68,-3.35)	513.15(468.75,557.55)	-3.60(-4.03,-3.17)
Protein-energy malnutrition	68.02(52.24,89.10)	-10.76(-11.67,-9.85)	121.08(76.28,178.24)	-15.62(-16.38,-14.85)	30.81(19.93,41.69)	-4.33(-4.95,-3.71)	38.85(26.63,51.06)	-1.34(-1.73,-0.95)	63.93(48.26,79.60)	-1.66(-2.27,-1.05)	291.16(257.71,324.60)	-3.52(-4.29,-2.74)
Iodine deficiency	27.72(12.79,54.67)	-1.68(-1.92,-1.45)	0.32(0.08,0.75)	-8.79(-10.16,-7.39)	4.18(3.44,5.08)	-7.78(-8.98,-6.56)	39.60(27.27,51.93)	-1.38(-1.65,-1.12)	36.73(24.85,48.61)	-1.05(-1.37,-0.73)	25.84(15.88,35.81)	-1.05(-1.36,-0.73)
Vitamin A deficiency	3.93(2.34,6.30)	-5.65(-5.87,-5.42)	12.64(6.71,22.17)	-6.77(-7.03,-6.50)	6.81(5.85,7.94)	-6.90(-7.11,-6.68)	2.11(1.60,2.77)	-0.60(-0.87,-0.32)	1.62(1.16,2.28)	1.38(1.09,1.68)	1.55(0.88,2.73)	1.03(0.74,1.31)

Dietary iron deficiency	73.47(44.65,115.33)	-5.47(-5.63,-5.32)	117.86(53.25,220.45)	-5.19(-5.46,-4.91)	58.23(43.28,73.19)	-5.68(-5.85,-5.50)	59.34(44.24,74.43)	-5.39(-5.55,-5.23)	96.21(76.98,115.43)	-5.34(-5.48,-5.20)	101.80(82.03,121.58)	-6.15(-6.38,-5.91)
Other nutritional deficiency	27.92(21.31,36.26)	-0.82(-1.53,-0.10)	59.87(34.05,101.19)	-4.16(-5.29,-3.01)	9.33(8.19,10.62)	0.66(0.25,1.06)	18.48(16.85,20.26)	1.86(1.29,2.43)	27.73(17.41,38.06)	1.70(0.85,2.56)	92.80(73.92,111.68)	2.89(2.11,3.68)
**Hainan**												
Overall nutritional deficiencies	312.91(238.58,413.70)	-6.64(-7.05,-6.22)	673.37(463.33,920.57)	-9.92(-10.38,-9.46)	137.52(114.54,160.51)	-5.56(-5.91,-5.21)	206.45(178.29,234.62)	-3.40(-3.57,-3.24)	307.56(273.19,341.94)	-3.51(-3.76,-3.26)	964.54(903.67,1025.41)	-4.02(-4.58,-3.45)
Protein-energy malnutrition	146.16(118.82,176.09)	-8.50(-9.11,-7.89)	422.30(279.34,614.81)	-11.22(-11.74,-10.69)	40.74(28.23,53.25)	-5.68(-6.36,-5.00)	53.39(39.07,67.71)	-2.95(-3.48,-2.43)	106.18(85.98,126.37)	-3.23(-3.92,-2.53)	716.09(663.64,768.54)	-4.06(-4.80,-3.31)
Iodine deficiency	35.43(16.09,71.00)	0.76(0.20,1.32)	0.38(0.11,0.91)	-3.73(-4.40,-3.06)	5.16(3.59,7.41)	-2.80(-3.21,-2.39)	50.54(36.60,64.47)	0.85(0.26,1.44)	47.16(33.70,60.62)	1.07(0.43,1.71)	33.64(22.27,45.01)	1.07(0.48,1.67)
Vitamin A deficiency	3.55(2.15,5.43)	-4.71(-5.01,-4.41)	12.65(6.43,21.27)	-5.35(-5.64,-5.06)	5.81(4.13,8.18)	-6.03(-6.31,-5.74)	1.77(1.03,3.07)	-1.11(-1.66,-0.56)	1.40(0.70,2.78)	0.67(0.49,0.85)	1.43(0.40,5.14)	0.30(0.20,0.41)

Dietary iron deficiency	102.32(60.36,159.80)	-5.25(-5.52,-4.98)	186.35(82.34,350.61)	-4.85(-5.12,-4.57)	78.05(60.73,95.36)	-5.80(-6.10,-5.50)	82.12(64.36,99.89)	-5.24(-5.54,-4.95)	123.35(101.58,145.12)	-4.98(-5.19,-4.76)	144.13(120.60,167.66)	-5.49(-5.81,-5.18)
Other nutritional deficiency	25.46(18.46,34.73)	0.09(-0.50,0.68)	51.70(26.93,95.65)	-0.69(-1.99,0.62)	7.76(5.78,10.43)	-0.60(-1.01,-0.19)	18.63(15.73,22.05)	0.38(-0.31,1.06)	29.48(18.84,40.13)	0.48(-0.55,1.53)	69.25(52.94,85.56)	1.95(1.24,2.66)
**Hebei**												
Overall nutritional deficiencies	174.01(116.82,253.14)	-3.37(-3.59,-3.15)	217.19(134.35,337.37)	-6.27(-6.73,-5.82)	115.54(94.47,136.61)	-3.93(-4.23,-3.63)	161.32(136.43,186.22)	-2.23(-2.40,-2.05)	218.42(189.45,247.38)	-2.33(-2.45,-2.20)	249.37(218.42,280.33)	-3.16(-3.44,-2.88)
Protein-energy malnutrition	41.02(27.17,59.41)	-3.44(-3.84,-3.04)	62.22(41.55,91.38)	-8.61(-9.29,-7.91)	31.90(20.83,42.97)	0.63(0.31,0.95)	33.83(22.43,45.23)	1.21(1.04,1.39)	42.27(29.52,55.01)	1.00(0.87,1.13)	72.59(55.89,89.29)	-1.46(-1.84,-1.07)
Iodine deficiency	15.54(7.15,30.44)	0.70(0.15,1.25)	0.22(0.05,0.53)	-2.84(-3.38,-2.30)	2.78(2.33,3.31)	-2.29(-2.62,-1.95)	21.96(12.77,31.14)	0.76(0.18,1.35)	20.75(11.82,29.68)	1.05(0.44,1.66)	14.60(12.87,16.56)	1.12(0.53,1.71)
Vitamin A deficiency	2.91(1.77,4.44)	-4.33(-4.67,-3.98)	11.11(6.00,18.17)	-4.91(-5.35,-4.47)	4.52(3.94,5.18)	-5.85(-6.19,-5.51)	1.24(0.97,1.58)	-0.95(-1.12,-0.78)	1.45(1.16,1.82)	0.70(0.49,0.92)	1.58(1.08,2.32)	0.53(0.36,0.69)

Dietary iron deficiency	100.10(61.56,154.25)	-4.00(-4.26,-3.74)	131.17(59.16,242.44)	-4.73(-5.17,-4.29)	67.95(51.80,84.11)	-4.97(-5.32,-4.62)	88.78(70.31,107.25)	-3.61(-3.89,-3.33)	136.87(113.94,159.80)	-3.41(-3.59,-3.23)	142.37(118.98,165.75)	-4.29(-4.64,-3.93)
Other nutritional deficiency	14.45(9.06,21.63)	0.16(-0.17,0.49)	12.47(7.55,21.13)	-2.39(-3.40,-1.37)	8.39(7.59,9.28)	0.37(0.09,0.65)	15.51(14.48,16.62)	0.81(0.27,1.35)	17.08(15.99,18.24)	0.76(-0.22,1.74)	18.23(16.29,20.40)	0.64(0.14,1.14)
**Heilongjiang**												
Overall nutritional deficiencies	196.76(127.41,287.67)	-3.05(-3.24,-2.86)	215.58(125.87,352.33)	-6.62(-7.22,-6.01)	134.04(111.35,156.73)	-3.31(-3.51,-3.10)	194.00(166.70,221.30)	-1.71(-1.84,-1.57)	227.72(198.15,257.30)	-2.19(-2.31,-2.08)	275.76(243.21,308.30)	-2.96(-3.24,-2.67)
Protein-energy malnutrition	49.11(29.74,76.43)	-3.41(-3.80,-3.02)	51.02(35.13,70.44)	-9.93(-10.76,-9.09)	40.74(28.23,53.25)	0.21(-0.35,0.78)	43.40(30.48,56.31)	0.96(0.60,1.31)	52.56(38.35,66.77)	0.81(0.73,0.88)	92.04(73.23,110.84)	-1.50(-1.86,-1.14)
Iodine deficiency	25.58(11.63,50.36)	0.81(0.25,1.36)	0.31(0.06,0.74)	-2.85(-3.60,-2.09)	3.97(3.02,5.22)	-2.20(-2.69,-1.71)	36.39(24.56,48.21)	0.86(0.27,1.44)	34.17(22.72,45.63)	1.13(0.49,1.77)	24.00(14.40,33.61)	1.31(0.71,1.90)
Vitamin A deficiency	3.91(2.37,6.04)	-3.28(-3.73,-2.82)	13.26(7.08,22.73)	-3.90(-4.49,-3.32)	6.20(4.98,7.72)	-4.45(-4.88,-4.01)	2.13(1.65,2.75)	-1.44(-1.91,-0.97)	1.68(1.29,2.19)	0.60(0.25,0.95)	1.53(0.90,2.60)	0.32(0.01,0.62)

Dietary iron deficiency	99.98(59.66,157.90)	-3.83(-4.08,-3.58)	141.14(61.48,269.63)	-4.17(-4.59,-3.76)	72.61(55.90,89.31)	-4.44(-4.76,-4.11)	92.07(73.26,110.88)	-3.36(-3.56,-3.15)	117.25(96.03,138.48)	-3.79(-4.00,-3.58)	130.95(108.52,153.38)	-4.53(-4.89,-4.16)
Other nutritional deficiency	18.19(10.76,29.04)	-0.30(-0.75,0.15)	9.85(6.24,15.61)	-5.34(-5.80,-4.87)	10.52(8.89,12.45)	-0.25(-0.74,0.24)	20.01(11.24,28.78)	0.61(0.01,1.20)	22.06(12.86,31.27)	0.62(-0.32,1.57)	27.24(17.01,37.47)	0.90(0.33,1.47)
**Henan**												
Overall nutritional deficiencies	123.39(80.02,179.35)	-5.08(-5.40,-4.75)	166.39(90.68,294.57)	-8.72(-9.51,-7.93)	97.77(78.39,117.15)	-4.52(-4.77,-4.27)	105.72(85.57,125.87)	-3.65(-3.82,-3.48)	148.65(124.75,172.54)	-3.77(-3.96,-3.58)	193.74(166.46,221.03)	-4.29(-4.60,-3.98)
Protein-energy malnutrition	27.12(16.99,41.28)	-6.50(-7.43,-5.56)	27.09(18.48,38.42)	-13.81(-14.91,-12.69)	23.21(13.77,32.65)	0.07(-0.24,0.37)	23.50(14.00,33.00)	1.33(1.15,1.51)	28.50(18.04,38.97)	0.94(0.79,1.09)	55.36(40.78,69.95)	-2.35(-2.76,-1.93)
Iodine deficiency	4.37(1.97,8.80)	-3.15(-3.85,-2.44)	0.10(0.02,0.25)	-4.56(-5.93,-3.16)	1.12(0.89,1.41)	-4.65(-5.99,-3.28)	6.07(5.50,6.68)	-3.08(-3.73,-2.43)	5.76(5.19,6.39)	-2.95(-3.62,-2.27)	4.14(3.36,5.09)	-3.02(-3.80,-2.24)
Vitamin A deficiency	4.13(2.47,6.34)	-3.90(-4.31,-3.49)	13.46(7.64,21.74)	-4.84(-5.36,-4.32)	6.42(5.84,7.06)	-5.39(-5.76,-5.01)	2.33(1.99,2.73)	-0.70(-1.01,-0.38)	1.91(1.59,2.29)	1.11(0.82,1.39)	1.88(1.39,2.56)	0.78(0.53,1.03)

Dietary iron deficiency	77.35(46.50,122.88)	-5.03(-5.29,-4.76)	118.54(51.19,239.40)	-5.03(-5.42,-4.64)	60.94(45.64,76.24)	-5.37(-5.66,-5.08)	62.91(47.37,78.46)	-4.88(-5.17,-4.60)	100.50(80.85,120.15)	-4.79(-5.01,-4.56)	114.09(93.15,135.02)	-5.46(-5.82,-5.09)
Other nutritional deficiency	10.41(6.51,16.01)	-0.21(-0.63,0.21)	7.21(4.16,11.91)	-5.92(-6.39,-5.45)	6.07(5.51,6.70)	0.63(0.27,0.99)	10.91(10.15,11.73)	1.28(0.72,1.84)	11.98(11.14,12.87)	1.39(0.42,2.37)	18.27(16.55,20.17)	2.36(1.68,3.05)
**Hong Kong Special Administrative Region of China**												
Overall nutritional deficiencies	200.54(129.07,295.51)	-1.68(-1.90,-1.47)	175.80(83.08,349.45)	-2.66(-2.97,-2.35)	168.50(143.06,193.94)	-1.37(-1.60,-1.15)	197.81(170.24,225.38)	-1.34(-1.54,-1.15)	238.02(207.78,268.26)	-1.61(-1.84,-1.38)	252.79(221.63,283.95)	-2.66(-2.98,-2.34)
Protein-energy malnutrition	62.22(38.37,93.26)	-0.94(-1.26,-0.63)	17.10(11.50,24.41)	-6.21(-6.78,-5.63)	63.10(47.53,78.67)	1.08(0.57,1.59)	62.61(47.10,78.12)	0.03(-0.33,0.40)	74.23(57.34,91.12)	-0.92(-1.18,-0.66)	92.67(73.80,111.54)	-2.64(-3.01,-2.26)
Iodine deficiency	5.30(2.41,10.66)	-1.38(-1.44,-1.33)	0.11(0.00,0.28)	-3.55(-4.28,-2.81)	1.24(0.43,3.52)	-3.31(-3.86,-2.76)	7.47(5.41,10.30)	-1.32(-1.40,-1.24)	6.85(5.02,9.35)	-1.11(-1.21,-1.01)	4.81(2.64,8.76)	-1.10(-1.19,-1.01)

Vitamin A deficiency	2.92(1.63,4.91)	-0.68(-0.88,-0.48)	10.31(5.24,17.62)	-1.13(-1.31,-0.94)	4.91(2.91,8.29)	-1.40(-1.64,-1.17)	1.13(0.49,2.58)	0.84(0.62,1.05)	1.88(1.04,3.40)	1.37(1.22,1.51)	1.76(0.65,4.74)	0.62(0.51,0.73)
Dietary iron deficiency	105.50(60.59,171.95)	-2.24(-2.46,-2.02)	145.34(56.81,310.42)	-1.90(-2.13,-1.67)	83.32(65.43,101.21)	-2.50(-2.75,-2.25)	97.59(78.23,116.95)	-2.15(-2.36,-1.94)	123.88(102.07,145.70)	-2.04(-2.25,-1.83)	121.80(100.17,143.43)	-3.08(-3.41,-2.75)
Other nutritional deficiency	24.60(15.18,36.89)	-0.50(-1.21,0.22)	2.94(1.53,4.92)	-0.67(-1.73,0.41)	15.94(11.91,21.32)	0.27(-0.22,0.76)	29.01(18.46,39.57)	-0.28(-0.98,0.42)	31.17(20.23,42.12)	-1.15(-2.19,-0.11)	31.75(20.71,42.79)	-0.99(-1.66,-0.31)
**Hubei**												
Overall nutritional deficiencies	154.69(105.30,217.39)	-5.55(-5.95,-5.15)	217.87(134.42,347.91)	-8.94(-9.49,-8.38)	105.68(85.53,125.83)	-4.89(-5.18,-4.60)	129.58(107.27,151.89)	-4.32(-4.55,-4.09)	180.07(153.77,206.38)	-4.16(-4.46,-3.86)	302.67(268.57,336.77)	-4.29(-4.78,-3.80)
Protein-energy malnutrition	37.88(26.52,54.49)	-6.98(-7.87,-6.08)	42.63(28.56,62.16)	-13.44(-14.13,-12.74)	23.90(14.32,33.48)	-0.71(-0.98,-0.45)	28.20(17.79,38.60)	0.74(0.61,0.87)	39.42(27.11,51.72)	0.07(-0.31,0.46)	123.44(101.66,145.21)	-2.94(-3.76,-2.12)
Iodine deficiency	5.92(2.71,11.71)	-1.78(-2.44,-1.11)	0.11(0.02,0.32)	-4.17(-5.58,-2.74)	1.36(0.97,1.92)	-3.85(-5.14,-2.54)	8.26(7.38,9.25)	-1.68(-2.29,-1.06)	7.85(7.06,8.73)	-1.54(-2.20,-0.87)	5.63(4.18,7.58)	-1.69(-2.46,-0.90)

Vitamin A deficiency	5.56(3.24,8.82)	-3.26(-3.74,-2.78)	17.14(9.38,27.70)	-4.42(-4.99,-3.84)	8.46(7.38,9.71)	-4.66(-5.09,-4.22)	3.56(3.00,4.22)	-0.22(-0.63,0.19)	2.29(1.88,2.79)	0.95(0.66,1.23)	2.22(1.71,2.88)	0.72(0.48,0.97)
Dietary iron deficiency	89.56(54.46,139.98)	-5.67(-5.96,-5.39)	137.33(64.12,261.60)	-5.05(-5.42,-4.69)	65.52(49.65,81.38)	-5.85(-6.16,-5.53)	76.31(59.18,93.43)	-5.79(-6.05,-5.53)	113.19(92.34,134.04)	-5.44(-5.69,-5.18)	126.84(104.76,148.91)	-6.16(-6.52,-5.79)
Other nutritional deficiency	15.76(11.02,22.34)	0.07(-0.40,0.55)	20.66(11.76,35.10)	-3.66(-4.66,-2.64)	6.43(5.50,7.52)	0.91(0.46,1.36)	13.26(12.13,14.49)	1.62(1.05,2.19)	17.33(16.13,18.61)	1.55(0.64,2.47)	44.55(31.47,57.64)	2.71(2.10,3.31)
**Hunan**												
Overall nutritional deficiencies	147.62(101.60,205.48)	-4.82(-5.18,-4.46)	238.30(146.76,375.17)	-8.17(-8.77,-7.57)	108.09(87.71,128.47)	-4.57(-4.90,-4.23)	119.22(97.82,140.62)	-3.19(-3.33,-3.04)	165.37(140.17,190.58)	-3.36(-3.58,-3.14)	270.10(237.89,302.32)	-3.85(-4.37,-3.34)
Protein-energy malnutrition	39.21(25.50,56.08)	-5.78(-6.65,-4.90)	75.73(48.82,108.43)	-10.88(-11.93,-9.82)	26.49(16.40,36.57)	-0.39(-0.64,-0.13)	30.11(19.36,40.87)	1.09(1.00,1.18)	39.72(27.37,52.07)	0.54(0.29,0.78)	76.43(59.29,93.56)	-4.15(-4.99,-3.30)
Iodine deficiency	4.29(1.89,8.61)	-2.98(-3.62,-2.33)	0.09(0.02,0.24)	-4.49(-5.81,-3.16)	1.10(0.82,1.48)	-4.44(-5.70,-3.17)	5.96(5.29,6.72)	-2.93(-3.52,-2.33)	5.60(4.97,6.32)	-2.77(-3.37,-2.15)	4.04(3.19,5.13)	-2.71(-3.38,-2.02)

Vitamin A deficiency	5.23(3.07,8.33)	-3.38(-3.81,-2.95)	14.90(8.00,25.45)	-4.71(-5.24,-4.18)	8.30(7.45,9.25)	-4.67(-5.05,-4.29)	3.50(3.00,4.09)	-0.43(-0.81,-0.05)	2.08(1.71,2.54)	0.77(0.47,1.08)	1.96(1.39,2.76)	0.59(0.30,0.87)
Dietary iron deficiency	78.10(47.92,123.63)	-5.00(-5.25,-4.74)	123.85(53.22,238.14)	-5.22(-5.66,-4.78)	64.69(48.93,80.46)	-5.63(-5.98,-5.28)	64.53(48.78,80.27)	-4.66(-4.88,-4.43)	97.25(77.92,116.58)	-4.64(-4.85,-4.44)	100.87(81.19,120.56)	-5.63(-5.99,-5.27)
Other nutritional deficiency	20.78(15.84,27.81)	-2.01(-2.22,-1.81)	23.72(15.84,35.53)	-7.63(-8.49,-6.77)	7.51(6.71,8.41)	-0.28(-0.49,-0.08)	15.11(14.03,16.29)	1.12(0.67,1.58)	20.72(11.80,29.64)	0.89(0.15,1.63)	86.80(68.54,105.06)	1.62(1.15,2.08)
**Inner Mongolia**												
Overall nutritional deficiencies	185.10(124.68,267.29)	-3.57(-3.76,-3.37)	174.08(102.86,288.84)	-8.28(-8.89,-7.66)	129.13(106.86,151.40)	-3.74(-3.96,-3.52)	181.65(155.23,208.06)	-1.77(-1.98,-1.55)	228.22(198.61,257.83)	-2.08(-2.19,-1.97)	274.36(241.89,306.82)	-2.99(-3.21,-2.77)
Protein-energy malnutrition	55.92(34.15,82.82)	-4.57(-5.21,-3.93)	27.90(17.31,42.69)	-13.51(-14.51,-12.49)	46.29(32.96,59.63)	0.07(-0.38,0.52)	53.65(39.29,68.00)	0.84(0.49,1.18)	68.91(52.64,85.19)	0.49(0.25,0.74)	106.20(86.00,126.40)	-1.93(-2.33,-1.52)
Iodine deficiency	28.52(12.96,55.70)	0.24(-0.23,0.72)	0.33(0.10,0.80)	-4.50(-5.82,-3.17)	4.35(3.25,5.84)	-3.61(-4.60,-2.61)	40.49(28.02,52.97)	0.36(-0.15,0.87)	38.29(26.16,50.42)	0.62(0.07,1.18)	27.09(16.89,37.29)	0.73(0.22,1.24)

Vitamin A deficiency	3.04(1.83,4.66)	-4.28(-4.62,-3.93)	9.83(5.29,16.91)	-5.31(-5.73,-4.89)	4.52(3.38,6.03)	-5.96(-6.32,-5.60)	1.68(1.18,2.38)	-0.96(-1.26,-0.67)	1.72(1.23,2.41)	1.01(0.70,1.33)	1.73(0.88,3.40)	0.68(0.43,0.92)
Dietary iron deficiency	73.40(44.08,118.52)	-4.38(-4.60,-4.15)	115.73(54.15,232.29)	-4.31(-4.57,-4.04)	61.92(46.50,77.34)	-5.03(-5.29,-4.77)	60.96(45.66,76.27)	-4.08(-4.38,-3.78)	89.51(70.97,108.05)	-4.07(-4.25,-3.88)	96.45(77.20,115.69)	-5.12(-5.44,-4.79)
Other nutritional deficiency	24.23(15.74,35.03)	-0.50(-0.91,-0.09)	20.30(11.66,33.40)	-4.33(-5.26,-3.40)	12.05(10.10,14.38)	-0.23(-0.67,0.21)	24.87(15.09,34.64)	0.60(-0.07,1.26)	29.80(19.10,40.49)	0.42(-0.59,1.43)	42.89(30.05,55.73)	1.37(0.76,1.98)
**Jiangsu**												
Overall nutritional deficiencies	163.75(106.86,231.31)	-4.80(-5.17,-4.43)	174.58(98.90,299.96)	-8.26(-8.87,-7.64)	103.39(83.46,123.32)	-4.72(-5.05,-4.38)	146.37(122.65,170.08)	-3.94(-4.17,-3.71)	194.50(167.16,221.83)	-3.85(-4.18,-3.52)	343.02(306.72,379.32)	-4.01(-4.45,-3.56)
Protein-energy malnutrition	45.31(30.79,65.40)	-4.98(-5.81,-4.14)	30.44(21.08,43.18)	-12.83(-13.50,-12.15)	27.60(17.30,37.90)	-0.76(-1.12,-0.40)	34.24(22.77,45.70)	0.62(0.38,0.86)	47.19(33.73,60.66)	-0.22(-0.67,0.22)	168.87(143.40,194.34)	-2.78(-3.48,-2.07)
Iodine deficiency	8.24(3.78,16.79)	0.32(-0.14,0.77)	0.15(0.03,0.38)	-2.38(-2.94,-1.81)	1.73(1.33,2.24)	-2.04(-2.44,-1.65)	11.55(10.70,12.46)	0.36(-0.13,0.85)	11.02(10.21,11.88)	0.67(0.16,1.19)	7.74(6.72,8.92)	0.74(0.24,1.24)

Vitamin A deficiency	2.79(1.65,4.31)	-3.92(-4.35,-3.49)	10.35(5.47,17.24)	-4.43(-4.95,-3.92)	3.99(3.36,4.73)	-5.60(-6.03,-5.18)	1.34(1.07,1.67)	-1.35(-1.72,-0.97)	1.56(1.27,1.90)	0.44(0.24,0.63)	1.61(1.18,2.20)	0.07(-0.06,0.21)
Dietary iron deficiency	92.46(54.60,144.37)	-5.42(-5.72,-5.13)	126.39(55.60,244.52)	-5.03(-5.40,-4.66)	62.99(47.44,78.55)	-5.66(-6.00,-5.32)	83.73(65.80,101.67)	-5.43(-5.70,-5.17)	115.08(94.05,136.10)	-5.28(-5.58,-4.98)	134.20(111.49,156.90)	-5.82(-6.20,-5.44)
Other nutritional deficiency	14.94(9.34,23.02)	1.10(0.54,1.67)	7.24(4.33,11.77)	-3.21(-4.02,-2.39)	7.09(6.23,8.06)	0.77(0.39,1.16)	15.51(14.52,16.57)	1.70(1.08,2.32)	19.66(18.57,20.80)	1.73(0.71,2.75)	30.60(19.76,41.44)	1.90(1.15,2.66)
**Jiangxi**												
Overall nutritional deficiencies	184.17(124.32,265.18)	-5.61(-6.08,-5.13)	235.28(151.31,360.56)	-9.89(-10.64,-9.13)	116.18(95.06,137.31)	-5.13(-5.43,-4.84)	161.06(136.18,185.93)	-3.53(-3.69,-3.36)	217.74(188.82,246.66)	-3.72(-3.96,-3.47)	363.36(326.00,400.72)	-4.16(-4.69,-3.61)
Protein-energy malnutrition	56.07(40.41,76.42)	-7.59(-8.58,-6.58)	76.96(54.26,108.58)	-12.94(-13.75,-12.13)	32.22(21.09,43.34)	-2.89(-3.81,-1.96)	39.40(27.10,51.70)	-1.10(-1.86,-0.33)	58.65(43.64,73.66)	-1.54(-2.53,-0.54)	186.58(159.81,213.35)	-3.32(-4.32,-2.30)
Iodine deficiency	25.65(11.70,50.22)	-0.85(-1.21,-0.48)	0.31(0.08,0.73)	-6.29(-7.81,-4.74)	4.01(3.37,4.78)	-5.75(-7.07,-4.41)	36.53(24.68,48.37)	-0.61(-0.99,-0.23)	34.22(22.76,45.69)	-0.41(-0.81,-0.01)	23.82(14.26,33.39)	-0.48(-0.87,-0.09)

Vitamin A deficiency	3.58(2.21,5.55)	-4.79(-5.09,-4.49)	12.30(6.63,20.12)	-5.59(-5.96,-5.22)	5.51(4.74,6.39)	-6.40(-6.71,-6.08)	1.81(1.42,2.32)	-1.25(-1.60,-0.89)	1.91(1.46,2.49)	0.77(0.53,1.02)	1.94(1.22,3.08)	0.60(0.42,0.77)
Dietary iron deficiency	80.13(48.12,125.35)	-5.54(-5.79,-5.28)	126.40(56.97,247.16)	-5.23(-5.58,-4.88)	66.26(50.30,82.21)	-5.78(-6.07,-5.48)	65.59(49.71,81.46)	-5.48(-5.74,-5.22)	99.57(80.01,119.13)	-5.37(-5.59,-5.16)	111.01(90.36,131.66)	-6.15(-6.50,-5.79)
Other nutritional deficiency	18.74(12.67,26.47)	0.21(-0.41,0.84)	19.32(11.18,31.90)	-2.88(-3.69,-2.06)	8.19(7.25,9.25)	0.68(0.04,1.33)	17.73(16.38,19.18)	1.05(0.34,1.77)	23.40(13.92,32.88)	0.85(-0.15,1.86)	40.01(27.61,52.41)	2.15(1.41,2.90)
**Jilin**												
Overall nutritional deficiencies	164.04(109.58,236.52)	-3.90(-4.15,-3.65)	194.41(109.89,338.56)	-7.01(-7.55,-6.46)	110.47(89.87,131.07)	-4.32(-4.59,-4.05)	150.27(126.24,174.30)	-2.56(-2.70,-2.41)	194.64(167.30,221.99)	-2.94(-3.11,-2.77)	282.20(249.27,315.13)	-3.84(-4.21,-3.48)
Protein-energy malnutrition	45.98(31.03,65.48)	-4.44(-4.95,-3.92)	40.31(28.47,56.35)	-10.65(-11.38,-9.91)	29.86(19.15,40.56)	-1.14(-1.55,-0.72)	38.49(26.33,50.65)	-0.39(-0.68,-0.09)	55.16(40.60,69.71)	-0.88(-1.20,-0.55)	122.19(100.53,143.86)	-3.67(-4.23,-3.10)
Iodine deficiency	8.23(3.78,16.51)	0.17(-0.25,0.59)	0.15(0.02,0.37)	-2.64(-3.46,-1.80)	1.75(1.09,2.81)	-2.28(-2.90,-1.66)	11.54(10.10,13.18)	0.20(-0.25,0.65)	10.93(9.67,12.36)	0.57(0.06,1.07)	7.71(5.84,10.19)	0.77(0.29,1.25)

Vitamin A deficiency	3.57(2.10,5.60)	-3.54(-3.92,-3.16)	10.04(5.23,16.39)	-4.80(-5.23,-4.37)	5.43(4.15,7.10)	-5.14(-5.50,-4.78)	2.34(1.74,3.15)	-0.50(-0.89,-0.12)	1.81(1.34,2.44)	0.86(0.61,1.10)	1.74(0.97,3.13)	0.63(0.37,0.90)
Dietary iron deficiency	88.93(53.24,137.58)	-4.34(-4.58,-4.10)	134.74(56.60,270.22)	-4.68(-5.11,-4.25)	65.61(49.74,81.49)	-5.22(-5.56,-4.89)	80.31(62.75,97.88)	-3.79(-3.99,-3.59)	103.68(83.72,123.63)	-4.18(-4.37,-4.00)	112.18(91.42,132.94)	-5.05(-5.41,-4.69)
Other nutritional deficiency	17.33(11.63,25.07)	0.28(-0.29,0.84)	9.17(5.27,14.89)	-2.61(-3.59,-1.62)	7.82(6.25,9.78)	0.10(-0.43,0.63)	17.59(15.79,19.60)	0.68(0.01,1.35)	23.07(13.66,32.48)	0.37(-0.60,1.35)	38.37(26.23,50.51)	1.27(0.64,1.91)
**Liaoning**												
Overall nutritional deficiencies	161.96(104.92,233.81)	-3.80(-4.06,-3.54)	229.54(136.31,361.41)	-6.91(-7.48,-6.35)	104.39(84.36,124.41)	-3.87(-4.16,-3.57)	148.64(124.75,172.54)	-2.57(-2.71,-2.43)	185.21(158.53,211.88)	-2.60(-2.75,-2.44)	248.22(217.34,279.10)	-3.32(-3.64,-3.01)
Protein-energy malnutrition	33.88(23.01,47.89)	-5.10(-5.66,-4.53)	71.57(47.99,98.87)	-9.83(-10.58,-9.07)	19.35(17.26,21.69)	-0.54(-0.80,-0.29)	23.68(14.14,33.22)	1.69(1.50,1.87)	32.70(21.50,43.91)	1.33(1.26,1.40)	82.71(64.89,100.54)	-2.03(-2.39,-1.66)
Iodine deficiency	17.85(8.09,35.45)	0.74(0.18,1.30)	0.24(0.06,0.57)	-2.32(-2.80,-1.84)	3.10(2.33,4.12)	-1.81(-2.14,-1.48)	25.26(15.41,35.11)	0.79(0.20,1.38)	23.85(14.28,33.42)	1.02(0.41,1.64)	16.80(14.65,19.26)	1.20(0.61,1.78)

Vitamin A deficiency	2.56(1.56,4.00)	-2.71(-2.99,-2.42)	7.19(3.91,11.88)	-3.45(-3.80,-3.10)	4.00(3.11,5.14)	-4.29(-4.61,-3.96)	1.54(1.15,2.06)	-0.57(-0.84,-0.31)	1.53(1.19,1.97)	0.92(0.67,1.17)	1.52(0.97,2.40)	0.67(0.42,0.92)
Dietary iron deficiency	95.83(57.53,148.74)	-4.10(-4.37,-3.82)	136.73(57.03,257.71)	-3.49(-3.83,-3.16)	72.71(55.99,89.42)	-4.56(-4.89,-4.23)	87.20(68.89,105.50)	-4.04(-4.29,-3.79)	113.16(92.31,134.01)	-3.95(-4.19,-3.70)	121.31(99.72,142.89)	-4.77(-5.19,-4.34)
Other nutritional deficiency	11.83(7.98,16.92)	-1.17(-1.39,-0.96)	13.80(8.67,22.54)	-6.31(-6.87,-5.75)	5.24(4.21,6.52)	-0.16(-0.33,0.02)	10.97(9.83,12.23)	1.62(1.11,2.14)	13.97(12.84,15.19)	1.38(0.52,2.25)	25.88(15.91,35.85)	-0.04(-0.38,0.30)
**Macao Special Administrative Region of China**												
Overall nutritional deficiencies	178.37(118.76,255.20)	-2.30(-2.43,-2.16)	203.83(108.77,349.09)	-3.55(-3.84,-3.26)	120.61(99.09,142.14)	-2.50(-2.66,-2.34)	158.03(133.39,182.67)	-1.81(-1.91,-1.71)	217.70(188.78,246.62)	-1.78(-1.98,-1.59)	348.24(311.67,384.82)	-2.49(-2.68,-2.29)
Protein-energy malnutrition	55.05(39.07,74.46)	-2.11(-2.33,-1.90)	49.77(32.58,71.29)	-5.55(-6.11,-4.99)	31.16(20.22,42.10)	-0.44(-0.96,0.08)	41.86(29.18,54.54)	-0.35(-0.61,-0.10)	63.79(48.14,79.45)	-0.83(-0.99,-0.67)	192.81(165.60,220.03)	-2.02(-2.18,-1.87)

Iodine deficiency	5.00(2.31,9.80)	-1.60(-1.68,-1.51)	0.10(0.02,0.28)	-3.67(-4.34,-2.99)	1.21(0.05,31.68)	-3.33(-3.86,-2.79)	6.94(2.58,18.68)	-1.60(-1.72,-1.47)	6.69(2.26,19.85)	-1.21(-1.31,-1.12)	4.67(0.34,64.55)	-1.18(-1.31,-1.05)
Vitamin A deficiency	1.95(1.14,3.17)	-1.66(-1.93,-1.38)	6.60(3.21,11.35)	-1.87(-2.12,-1.63)	3.22(0.44,23.81)	-2.71(-2.99,-2.43)	0.82(0.05,14.64)	-0.46(-0.81,-0.10)	1.28(0.11,15.37)	0.78(0.57,0.99)	1.33(0.01,183.20)	0.33(0.18,0.49)
Dietary iron deficiency	100.67(57.91,160.16)	-2.62(-2.75,-2.49)	144.93(53.66,285.20)	-2.30(-2.46,-2.15)	77.23(60.01,94.46)	-3.08(-3.26,-2.89)	90.45(71.81,109.09)	-2.48(-2.58,-2.38)	123.13(101.38,144.88)	-2.38(-2.58,-2.19)	125.29(103.35,147.23)	-3.35(-3.63,-3.07)
Other nutritional deficiency	15.70(9.87,22.91)	-0.47(-1.14,0.20)	2.42(1.51,4.12)	-3.62(-4.11,-3.14)	7.79(2.15,28.19)	-0.69(-1.16,-0.21)	17.96(9.71,33.25)	-0.09(-0.77,0.60)	22.81(13.45,32.16)	-0.46(-1.48,0.57)	24.14(14.51,33.77)	-0.97(-1.50,-0.44)
**Ningxia**												
Overall nutritional deficiencies	174.89(117.35,250.94)	-4.52(-4.81,-4.23)	209.02(124.53,342.13)	-9.55(-10.09,-9.01)	118.95(97.57,140.32)	-4.01(-4.20,-3.82)	155.99(131.51,180.47)	-1.85(-1.98,-1.73)	211.24(182.76,239.73)	-2.22(-2.33,-2.12)	319.72(284.68,354.77)	-2.90(-3.17,-2.63)
Protein-energy malnutrition	54.66(37.29,77.83)	-6.49(-7.34,-5.63)	47.54(29.83,74.36)	-13.91(-14.61,-13.21)	36.39(24.57,48.22)	-0.28(-0.52,-0.05)	45.24(32.05,58.42)	1.08(0.95,1.21)	64.61(48.85,80.36)	0.63(0.34,0.92)	150.62(126.56,174.67)	-2.01(-2.50,-1.52)

Iodine deficiency	22.91(10.49,45.55)	0.02(-0.46,0.50)	0.28(0.07,0.66)	-4.60(-5.72,-3.46)	3.71(2.27,6.06)	-3.92(-4.85,-2.97)	32.62(21.42,43.81)	0.17(-0.32,0.67)	30.38(19.58,41.18)	0.36(-0.18,0.90)	21.45(12.37,30.53)	0.49(-0.02,1.00)
Vitamin A deficiency	3.62(2.11,5.60)	-4.45(-4.68,-4.23)	10.07(4.95,17.56)	-5.89(-6.13,-5.65)	5.62(3.77,8.37)	-5.96(-6.23,-5.69)	2.38(1.38,4.09)	-0.73(-1.06,-0.41)	1.78(0.86,3.69)	1.06(0.79,1.33)	1.68(0.40,7.11)	0.79(0.51,1.08)
Dietary iron deficiency	72.05(41.77,112.40)	-4.50(-4.73,-4.28)	131.13(56.49,255.68)	-4.42(-4.63,-4.20)	63.75(48.10,79.40)	-5.07(-5.29,-4.85)	55.01(40.47,69.54)	-4.17(-4.47,-3.87)	87.16(68.86,105.46)	-4.28(-4.49,-4.08)	98.44(78.99,117.88)	-5.22(-5.55,-4.90)
Other nutritional deficiency	21.65(14.59,30.99)	0.25(-0.24,0.74)	20.00(10.58,33.67)	-4.21(-5.23,-3.18)	9.48(6.97,12.88)	0.81(0.51,1.11)	20.76(11.83,29.69)	1.61(1.08,2.14)	27.31(17.07,37.55)	1.56(0.65,2.49)	47.54(34.03,61.06)	2.69(2.03,3.35)
**Qinghai**												
Overall nutritional deficiencies	189.86(127.14,270.26)	-3.97(-4.12,-3.82)	290.13(175.28,476.46)	-7.56(-8.07,-7.04)	139.78(116.60,162.95)	-3.72(-3.88,-3.57)	156.96(132.40,181.51)	-2.07(-2.31,-1.84)	221.38(192.22,250.54)	-2.16(-2.28,-2.04)	331.94(296.23,367.65)	-2.61(-2.79,-2.43)
Protein-energy malnutrition	48.38(33.65,67.30)	-6.36(-7.09,-5.62)	69.39(44.56,109.21)	-11.61(-12.37,-10.85)	30.74(19.88,41.61)	-2.25(-2.69,-1.80)	36.73(24.85,48.61)	-0.13(-0.49,0.24)	51.70(37.61,65.80)	-0.38(-0.73,-0.03)	130.67(108.26,153.07)	-2.05(-2.62,-1.47)

Iodine deficiency	15.90(7.21,31.16)	-0.32(-0.88,0.25)	0.22(0.05,0.55)	-4.09(-5.36,-2.82)	2.86(1.56,5.23)	-3.70(-4.89,-2.49)	22.48(13.19,31.78)	-0.19(-0.75,0.37)	21.08(12.08,30.08)	0.04(-0.52,0.61)	15.17(8.99,25.60)	0.15(-0.37,0.68)
Vitamin A deficiency	3.68(2.17,5.67)	-4.18(-4.33,-4.03)	11.56(6.09,19.97)	-5.06(-5.22,-4.91)	6.22(4.13,9.37)	-5.61(-5.91,-5.31)	2.03(1.10,3.74)	-0.36(-0.66,-0.06)	1.62(0.73,3.60)	1.33(1.08,1.58)	1.53(0.30,7.95)	0.94(0.69,1.18)
Dietary iron deficiency	101.56(60.74,156.34)	-3.47(-3.72,-3.21)	176.16(79.27,348.03)	-3.44(-3.60,-3.29)	91.41(72.67,110.15)	-4.03(-4.25,-3.81)	78.48(61.12,95.85)	-3.23(-3.59,-2.87)	124.10(102.27,145.94)	-3.12(-3.36,-2.87)	137.74(114.74,160.75)	-3.98(-4.23,-3.72)
Other nutritional deficiency	20.35(14.07,28.32)	0.06(-0.57,0.71)	32.81(14.99,61.12)	-2.37(-3.18,-1.54)	8.54(6.02,12.11)	0.26(-0.32,0.84)	17.23(13.97,21.26)	0.92(0.20,1.65)	22.86(13.49,32.23)	0.95(0.00,1.91)	46.82(33.41,60.23)	2.95(2.22,3.69)
**Shaanxi**												
Overall nutritional deficiencies	202.27(134.89,286.54)	-5.33(-5.65,-5.01)	248.53(149.55,389.27)	-9.85(-10.42,-9.29)	118.59(97.24,139.93)	-4.75(-4.98,-4.52)	183.49(156.94,210.04)	-3.28(-3.44,-3.12)	244.36(213.72,274.99)	-3.20(-3.34,-3.05)	388.68(350.04,427.32)	-3.50(-3.77,-3.22)
Protein-energy malnutrition	51.01(34.73,70.62)	-8.01(-8.87,-7.15)	54.79(35.73,79.20)	-14.35(-15.11,-13.58)	30.66(19.81,41.51)	-2.61(-3.04,-2.17)	39.29(27.00,51.57)	-0.52(-0.83,-0.21)	58.50(43.51,73.49)	-0.96(-1.37,-0.55)	159.14(134.41,183.87)	-3.34(-3.97,-2.72)

Iodine deficiency	26.95(12.33,52.53)	-0.75(-1.12,-0.38)	0.31(0.09,0.78)	-6.16(-7.30,-5.01)	4.15(3.28,5.24)	-5.48(-6.48,-4.46)	38.31(26.18,50.44)	-0.53(-0.92,-0.15)	36.08(24.31,47.86)	-0.37(-0.78,0.04)	25.42(15.54,35.30)	-0.37(-0.76,0.02)
Vitamin A deficiency	3.26(1.94,5.01)	-4.97(-5.28,-4.66)	9.95(5.56,17.15)	-6.20(-6.54,-5.87)	5.00(4.04,6.19)	-6.63(-6.96,-6.31)	1.95(1.50,2.53)	-0.65(-0.91,-0.39)	1.69(1.27,2.26)	1.19(0.90,1.49)	1.64(0.97,2.75)	0.90(0.59,1.21)
Dietary iron deficiency	98.62(59.04,156.10)	-4.96(-5.20,-4.72)	156.87(69.19,284.83)	-4.60(-4.89,-4.30)	70.17(53.75,86.58)	-5.32(-5.57,-5.07)	85.25(67.15,103.35)	-5.00(-5.27,-4.74)	121.45(99.85,143.05)	-4.67(-4.87,-4.48)	134.78(112.02,157.53)	-5.21(-5.50,-4.92)
Other nutritional deficiency	22.44(15.70,29.97)	-1.46(-1.97,-0.94)	26.60(14.71,44.53)	-5.79(-6.84,-4.72)	8.61(7.32,10.13)	-0.19(-0.73,0.36)	18.69(17.18,20.34)	0.61(-0.06,1.29)	26.63(16.51,36.74)	0.49(-0.43,1.43)	67.71(51.58,83.84)	2.04(1.34,2.75)
**Shandong**												
Overall nutritional deficiencies	159.08(100.77,239.26)	-3.61(-3.77,-3.46)	169.01(97.44,291.53)	-7.00(-7.58,-6.42)	117.27(96.05,138.50)	-4.27(-4.55,-3.99)	156.63(132.10,181.16)	-2.27(-2.46,-2.09)	186.80(160.01,213.58)	-2.76(-2.84,-2.68)	205.76(177.64,233.87)	-3.83(-4.05,-3.60)
Protein-energy malnutrition	33.25(18.56,56.67)	-3.95(-4.47,-3.43)	25.39(17.47,36.46)	-11.41(-12.46,-10.34)	35.49(23.81,47.17)	0.54(0.00,1.08)	31.96(20.88,43.04)	0.83(0.45,1.21)	32.45(21.29,43.62)	0.27(0.12,0.43)	44.92(31.79,58.06)	-3.31(-3.78,-2.83)

Iodine deficiency	25.68(11.92,49.47)	0.77(0.20,1.35)	0.30(0.08,0.75)	-3.15(-3.83,-2.46)	4.00(3.51,4.56)	-2.50(-2.94,-2.06)	36.38(24.56,48.20)	0.84(0.23,1.45)	34.68(23.14,46.22)	1.11(0.49,1.74)	24.35(14.68,34.02)	1.16(0.55,1.77)
Vitamin A deficiency	2.43(1.43,3.76)	-4.11(-4.52,-3.70)	7.26(3.77,12.25)	-5.42(-5.94,-4.90)	3.79(3.31,4.34)	-5.51(-5.90,-5.11)	1.34(1.09,1.64)	-0.84(-1.12,-0.57)	1.51(1.25,1.81)	0.94(0.65,1.24)	1.50(1.10,2.05)	0.72(0.48,0.96)
Dietary iron deficiency	83.63(49.95,134.84)	-4.53(-4.71,-4.34)	125.17(56.20,239.59)	-5.04(-5.46,-4.62)	64.89(49.10,80.68)	-5.45(-5.76,-5.13)	71.94(55.32,88.57)	-4.01(-4.20,-3.81)	103.87(83.89,123.84)	-4.17(-4.31,-4.03)	112.37(91.59,133.14)	-5.03(-5.32,-4.75)
Other nutritional deficiency	14.09(8.40,23.46)	-0.62(-1.03,-0.21)	10.88(6.67,17.00)	-4.42(-5.46,-3.37)	9.10(8.34,9.92)	-0.16(-0.64,0.32)	15.01(14.11,15.96)	0.34(-0.29,0.97)	14.29(13.46,15.18)	0.16(-0.77,1.10)	22.61(13.29,31.94)	0.84(0.30,1.38)
**Shanghai**												
Overall nutritional deficiencies	160.22(102.79,233.58)	-3.94(-4.36,-3.51)	148.11(78.02,263.20)	-4.56(-4.99,-4.14)	107.64(87.30,127.97)	-4.04(-4.52,-3.56)	165.14(139.95,190.33)	-3.65(-4.03,-3.26)	184.54(157.91,211.16)	-3.98(-4.42,-3.53)	223.83(194.51,253.16)	-4.40(-4.88,-3.92)
Protein-energy malnutrition	36.93(22.45,57.74)	-1.38(-1.64,-1.12)	23.96(16.12,33.67)	-6.72(-7.39,-6.05)	33.80(22.40,45.19)	0.48(0.10,0.86)	36.14(24.36,47.92)	0.34(0.04,0.65)	42.98(30.13,55.83)	0.04(-0.19,0.28)	53.35(39.03,67.66)	-2.46(-2.84,-2.08)

Iodine deficiency	4.47(2.01,8.89)	-1.16(-1.36,-0.97)	0.10(0.00,0.27)	-2.18(-2.78,-1.59)	1.14(0.61,2.15)	-2.04(-2.50,-1.58)	6.21(5.26,7.33)	-1.18(-1.39,-0.97)	5.86(4.86,7.07)	-0.98(-1.20,-0.76)	4.26(2.96,6.14)	-0.77(-0.98,-0.57)
Vitamin A deficiency	2.18(1.31,3.45)	-2.36(-2.76,-1.96)	5.73(3.06,9.27)	-2.97(-3.37,-2.56)	3.30(2.28,4.79)	-3.78(-4.30,-3.26)	1.36(0.96,1.94)	-1.02(-1.40,-0.65)	1.47(1.01,2.14)	0.52(0.35,0.68)	1.40(0.74,2.65)	0.22(0.09,0.36)
Dietary iron deficiency	102.27(61.06,162.06)	-4.81(-5.31,-4.31)	113.67(50.02,224.93)	-4.01(-4.47,-3.56)	60.78(45.50,76.06)	-5.34(-5.93,-4.75)	104.71(84.65,124.77)	-4.67(-5.14,-4.20)	116.10(94.98,137.22)	-5.07(-5.58,-4.55)	147.01(123.24,170.77)	-5.19(-5.73,-4.65)
Other nutritional deficiency	14.37(8.71,22.54)	-0.56(-1.11,0.00)	4.65(2.95,6.93)	-2.01(-3.25,-0.75)	8.62(6.85,10.85)	-0.51(-0.88,-0.13)	16.72(15.11,18.50)	-0.34(-0.98,0.31)	18.13(16.29,20.17)	-0.63(-1.63,0.38)	17.82(14.91,21.31)	-0.50(-1.07,0.07)
**Shanxi**												
Overall nutritional deficiencies	179.04(117.86,255.98)	-4.29(-4.55,-4.02)	216.49(127.96,364.14)	-7.06(-7.53,-6.59)	122.39(100.70,144.07)	-4.63(-4.94,-4.31)	165.19(140.00,190.38)	-3.20(-3.35,-3.05)	212.99(184.38,241.59)	-3.37(-3.60,-3.14)	293.26(259.69,326.82)	-3.85(-4.23,-3.47)
Protein-energy malnutrition	43.79(27.12,64.55)	-4.50(-5.04,-3.96)	39.37(26.65,55.24)	-11.11(-11.95,-10.27)	33.07(21.80,44.34)	-0.42(-0.86,0.02)	38.97(26.73,51.20)	0.44(0.11,0.77)	51.84(37.73,65.96)	0.10(-0.15,0.36)	90.80(72.12,109.47)	-2.98(-3.59,-2.37)

Iodine deficiency	12.96(5.90,25.41)	-0.20(-0.70,0.31)	0.19(0.04,0.44)	-3.92(-5.10,-2.72)	2.45(1.82,3.29)	-3.28(-4.28,-2.28)	18.30(16.82,19.92)	-0.05(-0.56,0.46)	17.21(15.67,18.90)	0.07(-0.48,0.62)	12.17(9.88,14.99)	-0.04(-0.61,0.53)
Vitamin A deficiency	2.09(1.22,3.35)	-5.53(-5.97,-5.10)	7.47(3.66,13.37)	-6.34(-6.80,-5.88)	3.60(2.82,4.59)	-6.89(-7.32,-6.45)	0.80(0.54,1.21)	-0.96(-1.29,-0.63)	1.19(0.83,1.69)	0.59(0.41,0.77)	1.30(0.69,2.47)	0.44(0.31,0.56)
Dietary iron deficiency	99.80(58.55,155.02)	-4.93(-5.22,-4.64)	147.55(66.23,291.62)	-5.01(-5.45,-4.56)	74.38(57.48,91.29)	-5.55(-5.93,-5.18)	88.69(70.23,107.15)	-4.65(-4.89,-4.41)	119.05(97.66,140.43)	-4.73(-5.00,-4.47)	136.50(113.60,159.40)	-5.35(-5.74,-4.96)
Other nutritional deficiency	20.40(13.87,28.71)	-0.58(-1.04,-0.13)	21.92(12.40,36.60)	-3.53(-4.69,-2.35)	8.89(7.61,10.39)	-0.32(-0.75,0.12)	18.42(16.93,20.05)	0.38(-0.25,1.02)	23.70(14.16,33.24)	0.33(-0.62,1.29)	52.49(38.29,66.69)	1.43(0.91,1.95)
**Sichuan**												
Overall nutritional deficiencies	153.66(101.88,216.77)	-5.40(-5.71,-5.09)	150.97(93.94,236.95)	-10.66(-11.14,-10.18)	99.10(79.59,118.61)	-4.17(-4.38,-3.95)	142.45(119.05,165.84)	-3.48(-3.67,-3.28)	196.52(169.05,224.00)	-3.53(-3.75,-3.31)	282.22(249.30,315.15)	-3.97(-4.33,-3.60)
Protein-energy malnutrition	54.29(36.13,79.99)	-6.57(-7.29,-5.85)	40.48(27.06,58.23)	-14.55(-15.28,-13.82)	40.31(27.87,52.76)	0.15(-0.13,0.44)	47.38(33.88,60.87)	1.19(1.01,1.37)	65.40(49.55,81.26)	0.76(0.52,1.00)	130.83(108.41,153.25)	-2.39(-2.98,-1.81)

Iodine deficiency	16.26(7.39,31.45)	-1.78(-2.23,-1.32)	0.22(0.05,0.56)	-6.36(-7.65,-5.05)	2.85(2.36,3.44)	-6.19(-7.47,-4.89)	23.09(13.67,32.51)	-1.52(-1.96,-1.09)	21.58(12.48,30.69)	-1.44(-1.89,-0.99)	14.97(13.52,16.58)	-1.68(-2.15,-1.20)
Vitamin A deficiency	1.80(1.04,2.90)	-6.86(-7.18,-6.53)	5.88(2.96,10.65)	-8.03(-8.30,-7.76)	3.44(2.90,4.09)	-7.78(-8.19,-7.37)	0.77(0.57,1.04)	-0.65(-1.00,-0.30)	0.80(0.61,1.07)	0.40(0.20,0.60)	0.90(0.60,1.37)	0.27(0.09,0.45)
Dietary iron deficiency	58.53(34.29,92.84)	-5.98(-6.20,-5.75)	85.58(35.70,167.78)	-5.00(-5.21,-4.78)	41.95(29.25,54.64)	-5.68(-5.92,-5.44)	49.28(35.52,63.04)	-6.22(-6.47,-5.96)	80.22(62.67,97.78)	-5.85(-6.05,-5.66)	82.91(65.07,100.76)	-6.76(-7.05,-6.48)
Other nutritional deficiency	22.79(15.61,33.48)	0.03(-0.37,0.43)	18.80(11.00,30.47)	-5.19(-6.29,-4.09)	10.55(9.57,11.63)	1.37(0.99,1.74)	21.92(12.75,31.10)	1.67(1.18,2.16)	28.51(18.04,38.98)	1.55(0.62,2.49)	52.61(38.39,66.83)	2.83(2.19,3.46)
**Tianjin**												
Overall nutritional deficiencies	147.38(93.31,216.69)	-3.02(-3.22,-2.82)	161.17(84.07,298.99)	-4.74(-5.16,-4.32)	106.47(86.25,126.70)	-3.49(-3.76,-3.22)	142.48(119.08,165.87)	-2.25(-2.40,-2.11)	177.30(151.20,203.40)	-2.69(-2.86,-2.52)	203.11(175.17,231.04)	-3.55(-3.87,-3.22)
Protein-energy malnutrition	37.40(22.61,58.42)	-1.42(-1.71,-1.12)	23.04(15.30,33.22)	-7.24(-8.04,-6.44)	31.06(20.14,41.99)	0.19(-0.29,0.67)	36.69(24.82,48.56)	0.59(0.22,0.96)	45.15(31.98,58.32)	0.29(0.03,0.55)	60.67(45.40,75.94)	-2.08(-2.42,-1.74)

Iodine deficiency	11.78(5.29,23.41)	0.43(-0.12,0.98)	0.19(0.00,0.51)	-2.70(-3.55,-1.84)	2.37(1.33,4.23)	-2.16(-2.72,-1.59)	16.37(14.27,18.78)	0.43(-0.16,1.02)	16.01(13.76,18.63)	0.87(0.24,1.50)	11.60(8.47,15.88)	1.13(0.55,1.72)
Vitamin A deficiency	2.24(1.31,3.51)	-2.77(-3.10,-2.44)	5.60(2.85,9.47)	-4.26(-4.64,-3.88)	3.32(2.04,5.42)	-4.25(-4.64,-3.87)	1.48(0.94,2.34)	-0.26(-0.49,-0.02)	1.50(0.91,2.46)	1.08(0.83,1.34)	1.47(0.61,3.56)	0.79(0.53,1.04)
Dietary iron deficiency	81.26(48.73,132.41)	-4.08(-4.38,-3.78)	127.79(54.93,256.36)	-4.06(-4.46,-3.66)	61.72(46.32,77.12)	-4.56(-4.94,-4.18)	71.00(54.49,87.52)	-3.73(-3.97,-3.48)	95.52(76.36,114.67)	-4.06(-4.31,-3.81)	108.46(88.05,128.87)	-4.80(-5.25,-4.34)
Other nutritional deficiency	14.70(8.80,23.15)	-0.34(-0.89,0.22)	4.55(2.91,7.33)	-2.57(-4.06,-1.05)	8.00(5.84,10.96)	-0.73(-1.17,-0.29)	16.93(14.79,19.37)	-0.04(-0.72,0.66)	19.12(16.65,21.96)	-0.18(-1.20,0.84)	20.91(11.94,29.87)	0.10(-0.47,0.67)
**Tibet**												
Overall nutritional deficiencies	447.58(323.48,624.50)	-4.65(-4.95,-4.35)	1187.45(819.59,1679.80)	-7.17(-7.72,-6.61)	370.51(332.78,408.24)	-3.16(-3.34,-2.98)	294.78(261.13,328.43)	-2.22(-2.41,-2.03)	397.29(358.22,436.36)	-2.31(-2.39,-2.23)	720.91(668.28,773.53)	-3.12(-3.49,-2.74)
Protein-energy malnutrition	87.50(66.02,112.67)	-9.40(-10.33,-8.47)	344.28(207.02,546.79)	-11.12(-11.99,-10.23)	34.18(22.72,45.64)	-7.38(-8.29,-6.46)	36.19(24.40,47.98)	-4.68(-5.38,-3.98)	59.44(44.33,74.55)	-4.56(-5.39,-3.71)	252.46(221.32,283.61)	-5.09(-6.06,-4.11)

Iodine deficiency	63.58(29.65,124.87)	-0.31(-0.68,0.07)	0.56(0.17,1.32)	-8.22(-9.79,-6.62)	7.96(5.18,12.24)	-6.15(-7.27,-5.02)	90.04(71.44,108.63)	-0.11(-0.52,0.30)	87.24(68.93,105.55)	0.26(-0.20,0.73)	63.38(47.78,78.99)	0.47(0.01,0.93)
Vitamin A deficiency	22.00(13.68,34.79)	-2.11(-2.32,-1.91)	78.54(48.35,122.44)	-2.68(-2.86,-2.49)	41.35(28.75,53.96)	-2.91(-3.09,-2.74)	11.50(8.17,16.17)	0.72(0.16,1.29)	3.16(1.28,7.83)	0.90(0.65,1.15)	3.13(0.49,19.91)	0.75(0.55,0.94)
Dietary iron deficiency	211.96(132.70,323.34)	-2.55(-2.77,-2.33)	453.38(233.07,782.41)	-1.89(-2.03,-1.76)	271.73(239.42,304.04)	-2.36(-2.54,-2.17)	136.25(113.37,159.13)	-2.84(-3.19,-2.49)	211.54(183.03,240.04)	-2.64(-2.83,-2.45)	244.35(213.71,274.98)	-3.21(-3.46,-2.96)
Other nutritional deficiency	62.54(46.90,83.73)	-1.21(-1.82,-0.60)	310.69(184.39,506.43)	-1.32(-2.11,-0.53)	15.29(11.21,20.85)	-2.33(-2.97,-1.69)	20.81(11.87,29.75)	-1.34(-1.72,-0.97)	35.92(24.17,47.66)	-0.64(-1.27,0.00)	157.58(132.98,182.19)	1.50(0.89,2.11)
**Xinjiang**												
Overall nutritional deficiencies	342.32(250.27,473.81)	-4.54(-4.67,-4.40)	835.91(581.38,1175.37)	-7.11(-7.27,-6.95)	155.27(130.85,179.70)	-4.65(-4.87,-4.43)	252.62(221.47,283.77)	-1.96(-2.15,-1.77)	331.13(295.47,366.80)	-2.17(-2.27,-2.07)	723.31(670.59,776.02)	-2.36(-2.67,-2.05)
Protein-energy malnutrition	131.43(103.96,163.97)	-6.57(-6.90,-6.25)	566.07(363.71,819.95)	-8.10(-8.29,-7.91)	37.92(25.85,49.98)	-5.42(-6.01,-4.84)	41.73(29.07,54.39)	-2.52(-2.87,-2.16)	75.98(58.90,93.07)	-1.88(-2.37,-1.39)	450.56(408.96,492.16)	-1.96(-2.53,-1.38)

Iodine deficiency	67.27(31.11,133.45)	0.80(0.25,1.34)	0.57(0.18,1.34)	-4.21(-5.17,-3.25)	8.24(6.89,9.85)	-3.07(-3.64,-2.49)	96.31(77.08,115.55)	0.86(0.30,1.42)	90.47(71.83,109.11)	1.16(0.50,1.82)	63.95(48.27,79.62)	1.30(0.70,1.91)
Vitamin A deficiency	3.06(1.88,4.63)	-4.17(-4.34,-4.00)	10.39(5.65,17.54)	-4.80(-5.01,-4.58)	5.04(4.01,6.33)	-5.42(-5.65,-5.20)	1.50(1.04,2.16)	-1.53(-1.76,-1.31)	1.53(1.01,2.33)	0.46(0.19,0.72)	1.52(0.65,3.53)	0.27(0.03,0.50)
Dietary iron deficiency	116.57(69.92,182.98)	-3.93(-4.08,-3.78)	183.66(80.73,338.16)	-4.17(-4.39,-3.96)	96.25(77.02,115.48)	-4.62(-4.79,-4.45)	97.22(77.90,116.55)	-3.55(-3.77,-3.33)	140.28(117.07,163.50)	-3.64(-3.81,-3.48)	161.11(136.23,185.98)	-4.33(-4.54,-4.13)
Other nutritional deficiency	23.98(16.62,33.41)	-0.11(-0.71,0.49)	75.22(37.49,136.73)	-0.34(-1.27,0.61)	7.83(6.52,9.40)	-0.78(-1.20,-0.37)	15.85(14.16,17.75)	0.21(-0.42,0.84)	22.86(13.49,32.24)	0.17(-0.79,1.14)	46.18(32.86,59.49)	1.08(0.49,1.67)
**Yunnan**												
Overall nutritional deficiencies	186.55(147.56,233.02)	-6.86(-7.34,-6.39)	283.51(201.81,389.41)	-12.01(-12.34,-11.68)	89.38(70.85,107.91)	-5.11(-5.36,-4.85)	113.43(92.56,134.31)	-3.06(-3.19,-2.94)	183.16(156.63,209.68)	-3.13(-3.33,-2.92)	804.06(748.49,859.64)	-1.40(-1.97,-0.83)
Protein-energy malnutrition	86.11(70.46,104.04)	-9.07(-9.97,-8.16)	118.94(79.42,171.14)	-14.58(-15.04,-14.11)	25.76(15.81,35.71)	-6.16(-6.92,-5.40)	35.45(23.78,47.12)	-2.77(-3.30,-2.24)	68.72(52.48,84.97)	-2.59(-3.34,-1.83)	574.03(527.07,620.99)	-1.23(-2.15,-0.31)

Iodine deficiency	8.88(4.06,17.72)	-2.80(-3.44,-2.16)	0.15(0.03,0.37)	-5.70(-6.99,-4.38)	1.86(1.42,2.42)	-6.00(-7.37,-4.61)	12.43(11.36,13.61)	-2.66(-3.26,-2.05)	11.85(10.65,13.18)	-2.44(-3.04,-1.85)	8.48(6.75,10.65)	-2.35(-2.95,-1.74)
Vitamin A deficiency	8.21(4.86,13.09)	-3.17(-3.50,-2.85)	26.13(14.43,41.35)	-3.96(-4.37,-3.56)	14.22(12.91,15.65)	-4.23(-4.48,-3.98)	5.07(4.41,5.84)	-0.77(-1.35,-0.19)	1.96(1.51,2.55)	0.30(0.01,0.60)	1.86(1.14,3.02)	0.22(-0.04,0.47)
Dietary iron deficiency	50.32(29.91,79.22)	-4.63(-4.87,-4.40)	68.01(25.84,143.68)	-4.88(-5.13,-4.62)	38.88(26.66,51.10)	-5.21(-5.40,-5.01)	43.17(30.30,56.05)	-4.11(-4.45,-3.76)	69.53(53.19,85.88)	-4.45(-4.69,-4.21)	64.96(49.16,80.76)	-5.92(-6.25,-5.60)
Other nutritional deficiency	33.03(26.48,42.41)	-1.41(-2.21,-0.61)	70.29(40.89,114.52)	-5.02(-6.22,-3.82)	8.66(7.66,9.80)	-0.58(-1.05,-0.10)	17.30(16.03,18.67)	0.77(0.12,1.43)	31.09(20.17,42.02)	1.13(0.25,2.01)	154.74(130.36,179.12)	3.67(2.63,4.73)
**Zhejiang**												
Overall nutritional deficiencies	204.00(134.64,294.93)	-3.81(-4.24,-3.38)	159.21(91.26,270.39)	-7.94(-8.49,-7.39)	141.52(118.20,164.84)	-3.77(-4.18,-3.36)	197.13(169.62,224.65)	-2.84(-3.17,-2.51)	261.04(229.37,292.71)	-2.81(-3.20,-2.42)	353.09(316.26,389.92)	-3.53(-4.01,-3.05)
Protein-energy malnutrition	83.33(50.77,126.68)	-1.92(-2.47,-1.37)	25.46(16.88,35.89)	-12.19(-12.76,-11.62)	64.27(48.55,79.98)	1.72(1.47,1.97)	79.75(62.24,97.25)	2.21(2.04,2.38)	106.72(86.47,126.97)	1.58(1.37,1.78)	186.49(159.73,213.26)	-1.73(-2.29,-1.16)

Iodine deficiency	8.12(3.75,16.27)	0.22(-0.23,0.67)	0.14(0.03,0.38)	-2.23(-2.83,-1.62)	1.76(1.30,2.37)	-1.92(-2.42,-1.42)	11.35(10.41,12.37)	0.24(-0.24,0.72)	10.85(9.91,11.88)	0.61(0.10,1.12)	7.65(6.35,9.22)	0.67(0.20,1.15)
Vitamin A deficiency	2.85(1.73,4.33)	-4.32(-4.77,-3.87)	8.35(4.45,13.88)	-5.66(-6.19,-5.12)	4.31(3.57,5.22)	-5.98(-6.45,-5.51)	1.74(1.40,2.17)	-0.92(-1.21,-0.62)	1.62(1.28,2.05)	0.92(0.66,1.19)	1.58(1.05,2.39)	0.80(0.54,1.05)
Dietary iron deficiency	77.34(47.20,122.31)	-6.01(-6.40,-5.63)	117.28(52.73,229.05)	-5.65(-6.16,-5.14)	54.93(40.40,69.45)	-6.36(-6.79,-5.92)	67.84(51.69,83.98)	-6.06(-6.42,-5.70)	97.25(77.92,116.58)	-5.67(-6.00,-5.33)	104.44(84.41,124.47)	-6.45(-6.90,-5.99)
Other nutritional deficiency	32.37(19.53,49.31)	1.61(1.16,2.07)	7.98(4.65,13.23)	-2.68(-3.82,-1.52)	16.25(14.73,17.93)	1.55(1.36,1.75)	36.46(24.63, 48.30)	2.03(1.56,2.50)	44.60(31.51,57.69)	1.69(0.77,2.63)	52.92(38.67, 67.18)	2.01(1.47,2.54)
